# Supplementary material for: SuperFi-Cas9 exhibits remarkable fidelity but severely reduced activity yet works effectively with ABE8e
Source: Nat Commun. 2022 Nov 11;13:6858. doi: 10.1038/s41467-022-34527-8 (PMC9652449; doi:10.1038/s41467-022-34527-8)
Supplement: Supplementary file 1 — Supplementary Information [file 41467_2022_34527_MOESM1_ESM.pdf]

# Summary

Supplementary Figures and legends ........ 2
Supplementary Figure 1 ........ 2
Supplementary Figure 2 ........ 4
Supplementary Figure 3 ........ 5
Supplementary Figure 4 ........ 6
Supplementary Figure 5 ........ 8
Supplementary Figure 6 ........ 9
Supplementary Figure 7 ........ 10
Supplementary Figure 8 ........ 12
Supplementary Figure 9 ........ 13
Supplementary Figure 10 ........ 15
Supplementary Figure 11 ........ 17
Supplementary Notes ........ 19
Supplementary Note 1: Cas9 spacer cloning ........ 19
Supplementary Note 2: SpCas9 variants, human expression plasmids ........ 19
pX330-Flag-WT SpCas9 (Addgene #126753) ........ 19
pX330-Flag-SpCas9-HF1 (Addgene #126755) ........ 23
pX330-Flag-HypaSpCas9 (Addgene #126756) ........ 25
pX330-Flag-evoSpCas9 (Addgene #126758) ........ 27
pPIK16045_pX330_Flag-SuperFi-Cas9 (Addgene #184370) ........ 29
px330-Flag-B-HypaR-SpCas9 (Addgene #126764) ........ 31
SuperFi mutants plasmids ........ 33
Supplementary Note 3: SpCas9 variants, bacterial expression plasmids ........ 34
Supplementary Note 4: Other plasmid sequences ........ 34
*Prnp*.HA-EGFP-DHFR[DD] ........ 34
*Sprn*.HA-CMV-EGFP ........ 37

# Supplementary Figures and legends

## Supplementary Figure 1

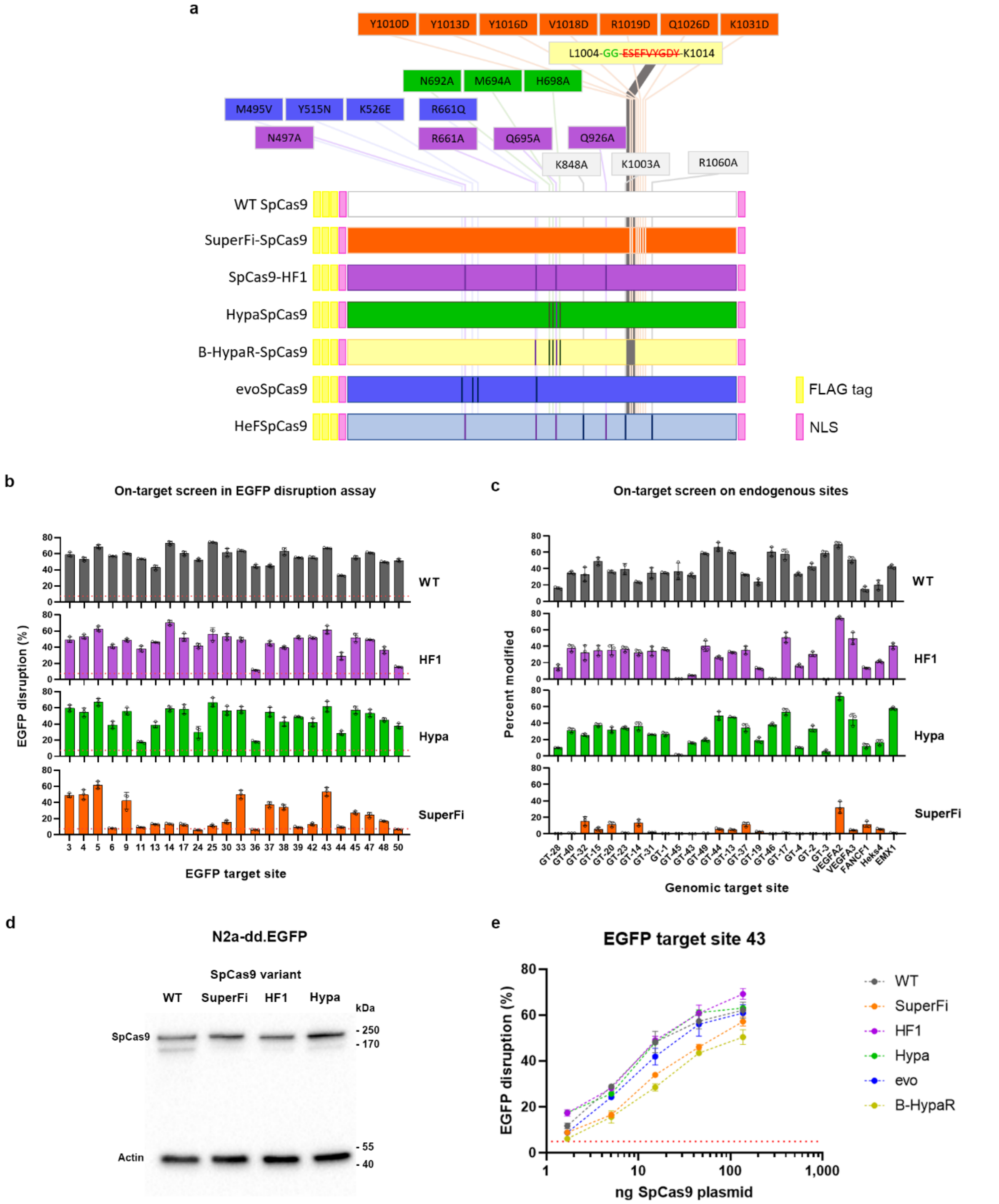


**Supplementary Figure 1. On-target activity of SuperFi-Cas9**

**a,** Schematic representation of the polypeptide chain of SpCas9s. Mutations of the IFNs used in this study are indicated on the linear sequence of SpCas9. **b,** On-target EGFP disruption data of WT SpCas9 and three IFN variants, as indicated in the panels, on 24 EGFP target sites. **c,** On-target activity of WT SpCas9 and three IFN variants, as indicated in the panels, across 26 endogenous sites as measured by NGS. **b-c,** Means are shown, error bars represent the standard deviation (SD) for triplicates (overlaid as white circles). **b,** Level of background EGFP

loss is indicated by a red dashed line (average of the percentage of dead SpCas9 controls from all target sites). (**c**) In case of endogenous target sites, the measured background level is available in Supplementary Data file 3. **d,** A single immunoblot analysis of the expression levels of SpCas9 nuclease variants in cell lysates of reporter N2a.dd-EGFP cells transfected with the indicated nuclease constructs. β-actin was used as a loading control for total protein amounts analyzed. **e,** Titration of expression plasmid amounts of wild-type SpCas9 and different IFN variants. Means are shown in case of each data point, error bars represent the standard deviation (SD) for triplicates; mean level of background EGFP loss in negative controls is represented by the red dashed line. **b-c, e**, Target sequences, EGFP disruption and NGS data are reported in Supplementary Data files 1–3.

## Supplementary Figure 2

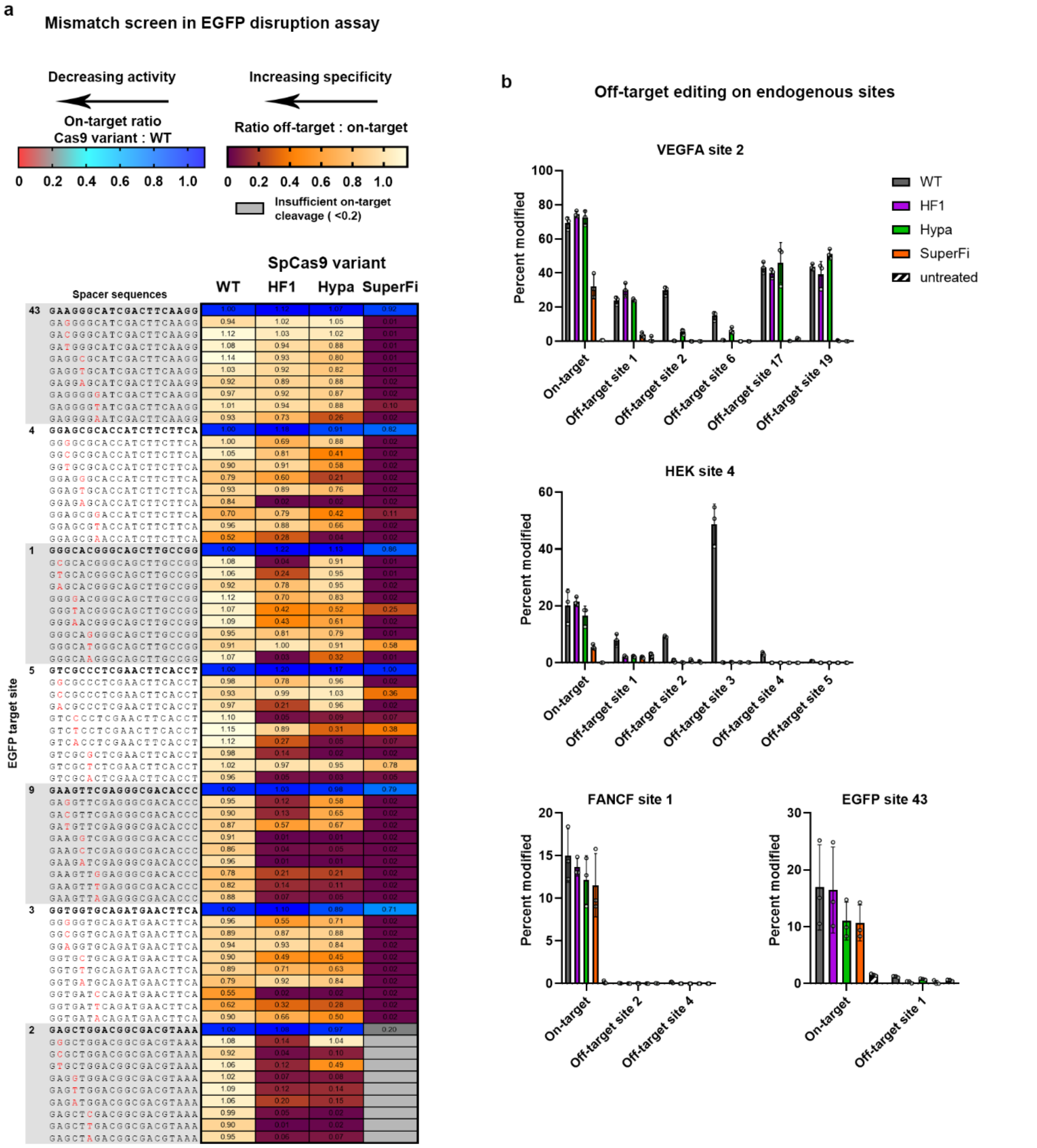


**Supplementary Figure 2. Mismatch tolerance of SuperFi-Cas9**

**a,** Mismatch screen of nuclease variants with either perfectly matching sgRNAs (red to blue) or single mismatching sgRNAs (yellow to brown) presented on a heatmap. Grey boxes: not determined due to low on-target activity (< 0.2 normalized to WT). **b,** On-target activity of WT SpCas9 and three IFN variants, as indicated in the panels, across 4 endogenous on-target sites with a total of 13 off-target sites as measured by NGS. Off-target sites were selected from previous GUIDE-seq experiments[11,24]. Means are shown, error bars represent the standard deviation (SD) for triplicates (overlaid as white circles). **a-b**, Target sequences, EGFP disruption and NGS data are reported in Supplementary Data files 1–3.

## Supplementary Figure 3

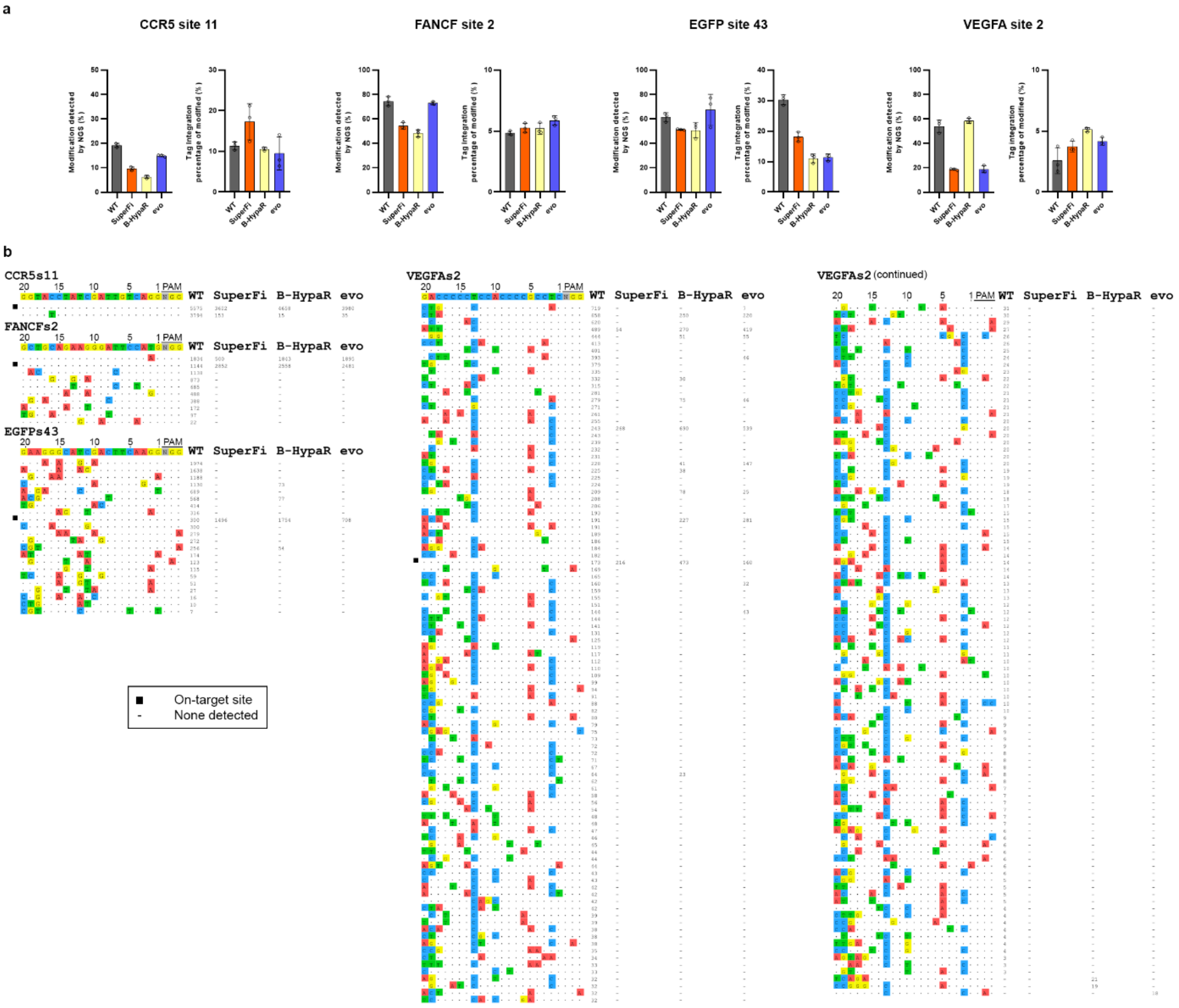


**Supplementary Figure 3. Genome-wide off-target effects of SuperFi-Cas9 variant identified by GUIDE-seq**

**a,** The percentage of on-target genome modification (indel + tag integration) and the tag integration frequency of the modified cells analyzed by NGS are presented in the bar charts. Means are shown, error bars represent the standard deviation (SD) for triplicates (overlaid as white circles). **b,** Off-target cleavage sites of SpCas9 variants identified by GUIDE-seq. Read counts represent a measure of cleavage frequency at a given site; mismatched positions within the spacer or PAM are highlighted in different colours. (-) indicates zero reads, which means that off-target cleavage was not detected, black squares indicate the on-target sites. **a-b,** Target sequences, NGS and GUIDE-seq data are available in Supplementary Data files 1, 3 and 4.

## Supplementary Figure 4

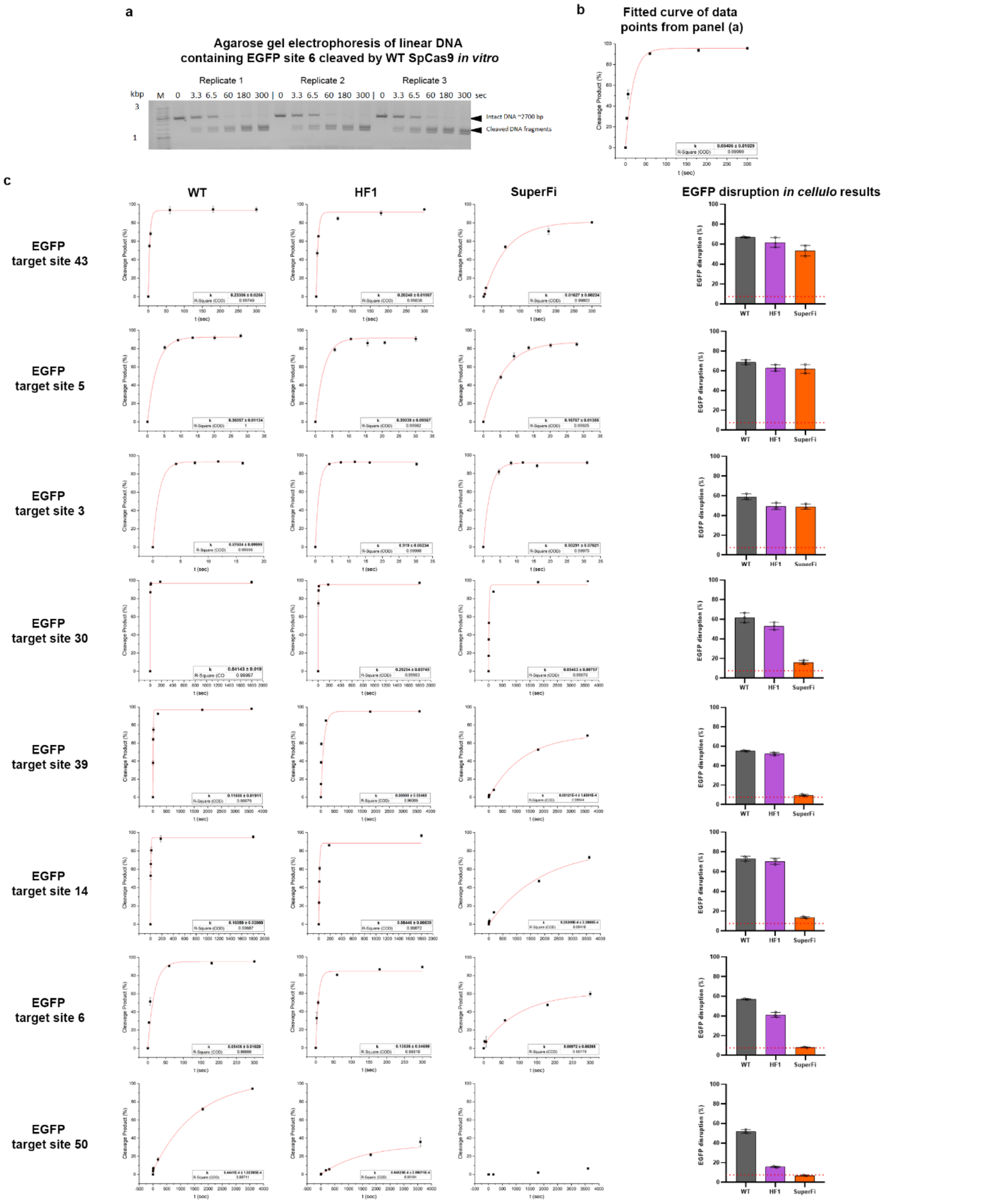


**Supplementary Figure 4. *In vitro* data-based fitted curves on 8 EGFP target sites cleaved with WT, -HF1 or SuperFi-SpCas9 variants**

**a,** Representative agarose gel showing the activity of WT SpCas9 in a linear plasmid-cleaving *in vitro* assay at different time points in triplicates. **b,** The plot shows the ratio of cleaved DNA, derived from the intensity of bands from the representative agarose gel in panel (a) (same as in panel [c]). **c,** Plots show the ratio of cleaved DNA, derived from the intensity of bands measured on agarose gel at different time points for all 8 target sites. Means with SD are shown; for n =3 replicates. Exponential curves were fitted to the values shown on the plot, *k* values were derived from these fitted curves. On the right side of the panel EGFP disruption data from Figure 1a (and

Supplementary Figure 1b) are shown for each target site. Summary of target and primer sequences and *in vitro* data are reported in Supplementary Data files 1 and 5.

**Supplementary Figure 5**

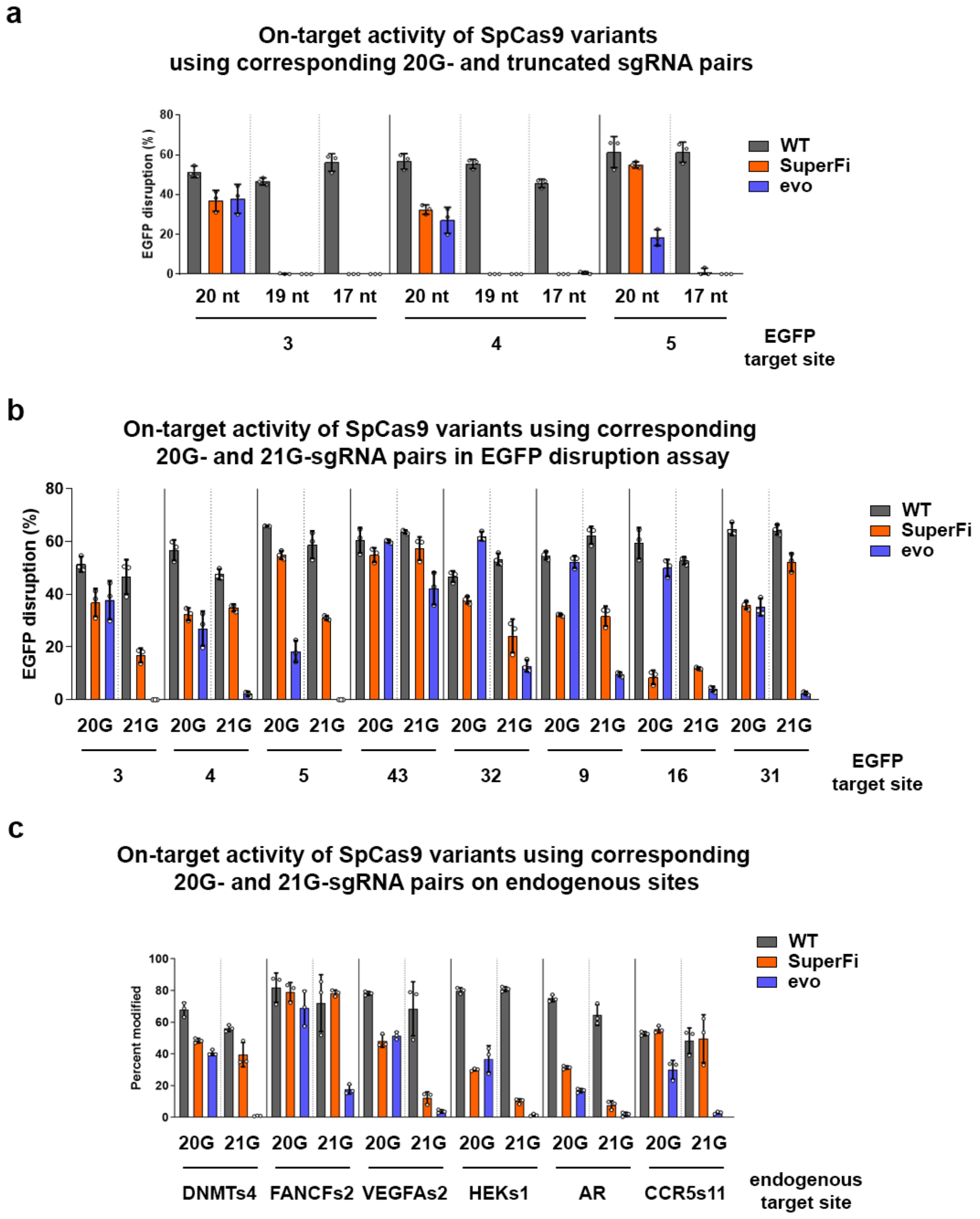


**Supplementary Figure 5. Tolerance of SuperFi-Cas9 to 5' modified sgRNAs**

Effects of (**a**) 5' truncation of sgRNAs and (**b, c**) 21G-sgRNAs on the activity of WT and three SpCas9 variants as indicated in the panels either (**a, b**) in EGFP disruption assay or (**c**) on endogenous target sites. Means are shown, error bars represent the standard deviation (SD) for triplicates (overlaid as white circles). Target sequences, EGFP disruption and NGS data are reported in Supplementary Data files 1, 2 and 3.

## Supplementary Figure 6

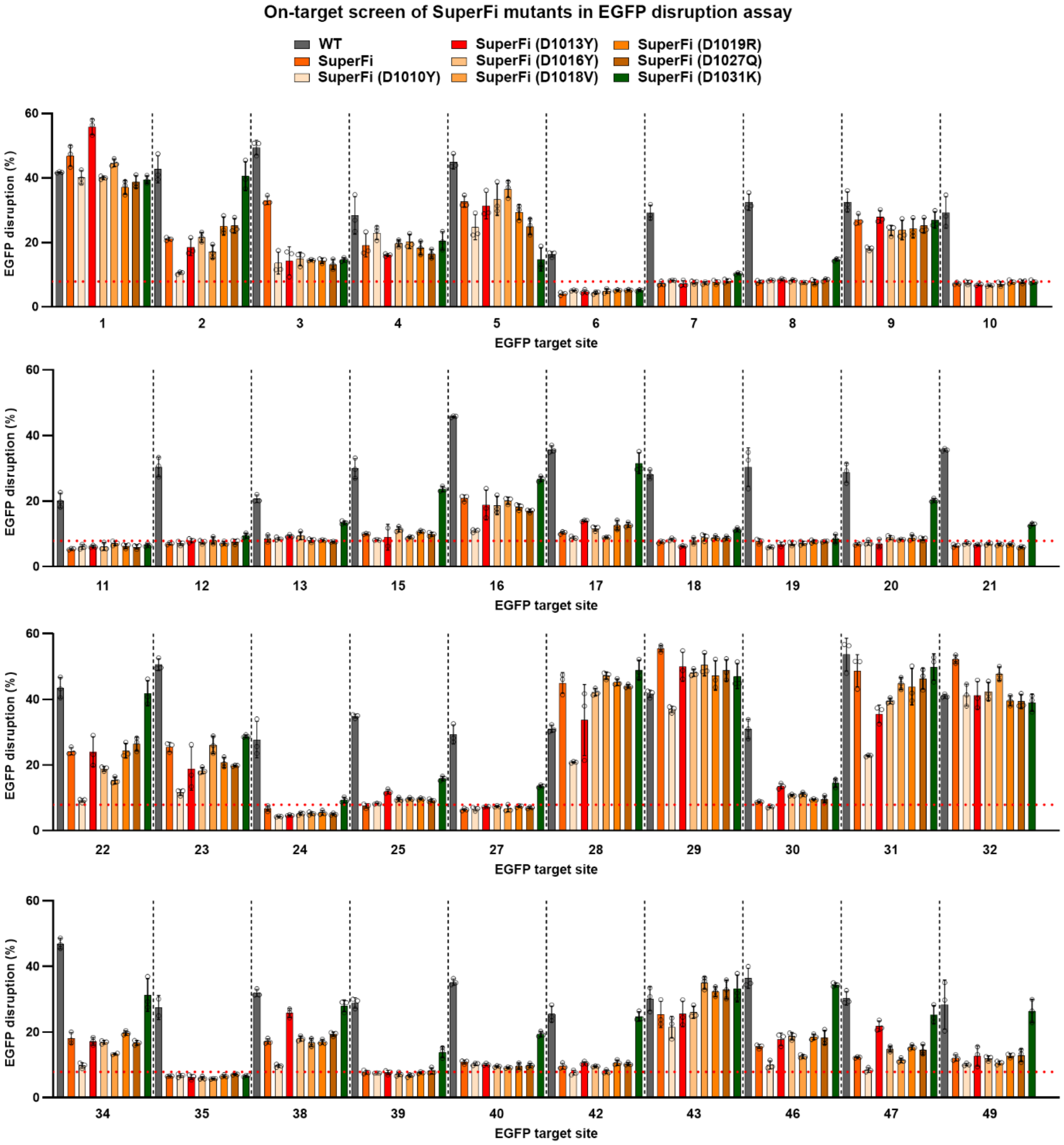


**Supplementary Figure 6. On-target activity of SuperFi variants, each lacking one mutation**

On-target EGFP disruption data on 40 EGFP target sites of WT SpCas9, SuperFi-Cas9 and seven SuperFi variants, each lacking one mutation as indicated in the figure. Means are shown, error bars represent the standard deviation (SD) for triplicates (overlaid as white circles). Level of background EGFP loss is indicated by a red dashed line (average of the percentage of dead SpCas9 controls from all target sites). Target sequences and EGFP disruption data are reported in Supplementary Data files 1 and 2.

## Supplementary Figure 7

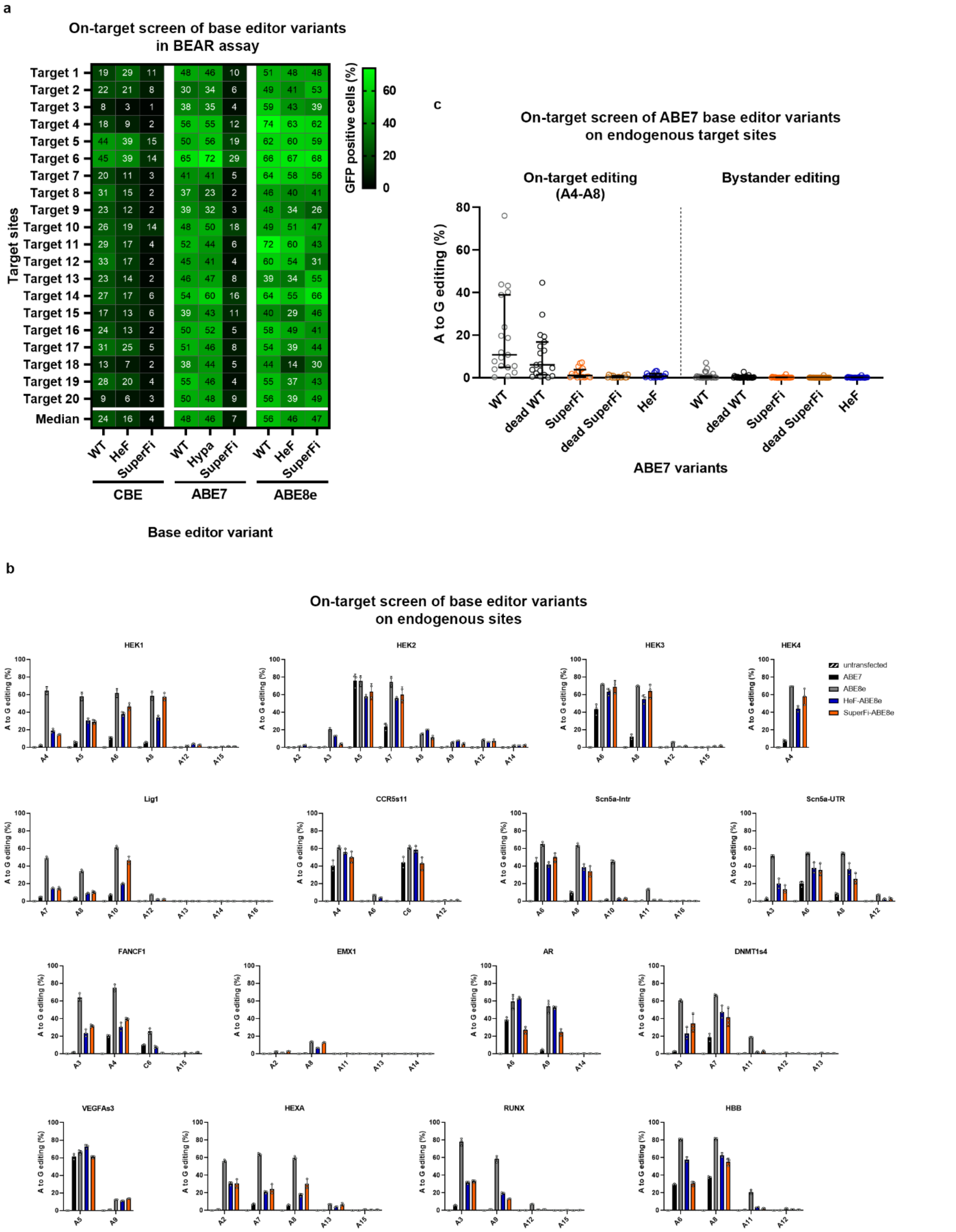


**Supplementary Figure 7. On-target activities of SuperFi base editor variants in the BEAR assay and on endogenous target sites**

**a**, On-target activity of WT and base editor variants as indicated in the panel. The heatmap shows mean on-target base editing activity (as GFP positive cells) of triplicates on 20 different target sites. **b,** Editing efficiencies of ABE7, WT-ABE8e, and two IFN ABE8e base editor variants are shown on 16 genomic loci for assessing on-target base

editing as measured by NGS. The diagrams show A to G (and in two cases C to G) conversion ratios at each adenine position for each target. As negative controls, base conversion for untransfected cells was measured (black with white dashes). Means are shown, error bars represent the standard deviation (SD) for triplicates (overlaid as white circles). **c,** Base editing activity of ABE7 variants on endogenous on-target sites, as measured by NGS. The editing efficiency of adenines inside (A4-A8, n=19) and outside the editing window (bystander editing, n=26) is shown side by side, separated with a dashed line. Results are presented on a scatter dot plot, the median and interquartile ranges are shown; data points are plotted as open circles representing the means of triplicates. Statistical significance was assessed by RM one-way ANOVA, statistical details and p-values are available in Methods and in Supplementary Data file 6. **a-c,** Target sequences, BEAR assay, NGS data and allele frequency tables are reported in Supplementary Data files 1–3 and 7.

## Supplementary Figure 8

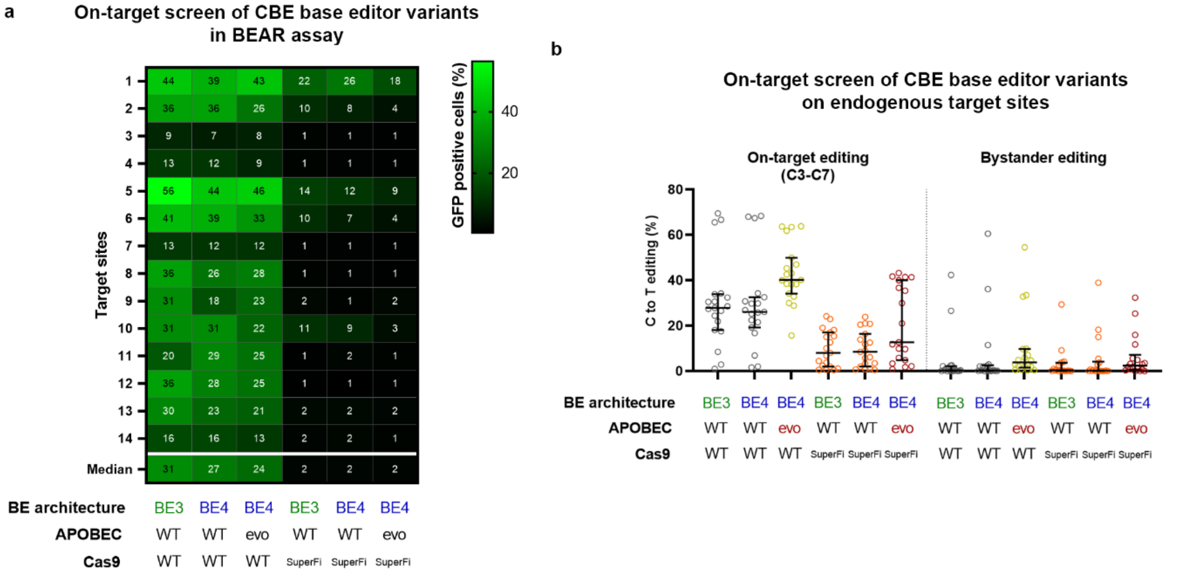


**Supplementary Figure 8. On-target activities of WT and SuperFi CBE base editor variants in the BEAR assay and on endogenous target sites**

**a-b,** Different WT SpCas9 and SuperFi CBE base editor variants were tested: CBE3 and CBE4max[44] (indicated as BE3 and BE4 in the figure, respectively) and evoCBE[34]. **a**, On-target activity of WT and base editor variants as indicated in the panel. The heatmap shows mean on-target base editing activity (as GFP positive cells) of triplicates on 14 different target sites. **b,** Base editing activity of CBE variants on endogenous on-target sites as measured by NGS. The editing efficiency of cytosines inside (C3-C7, n=19) and outside (bystander editing, n=18) the editing window is shown side by side, separated with a dashed line. Results are presented on a scatter dot plot, the median and interquartile ranges are shown; data points are plotted as open circles representing the means of triplicates. Statistical significance was assessed by RM one-way ANOVA, statistical details and p-values are available in Methods and in Supplementary Data file 6. **a-b,** Target sequences, BEAR assay and NGS data are reported in Supplementary Data files 1–3.

## Supplementary Figure 9

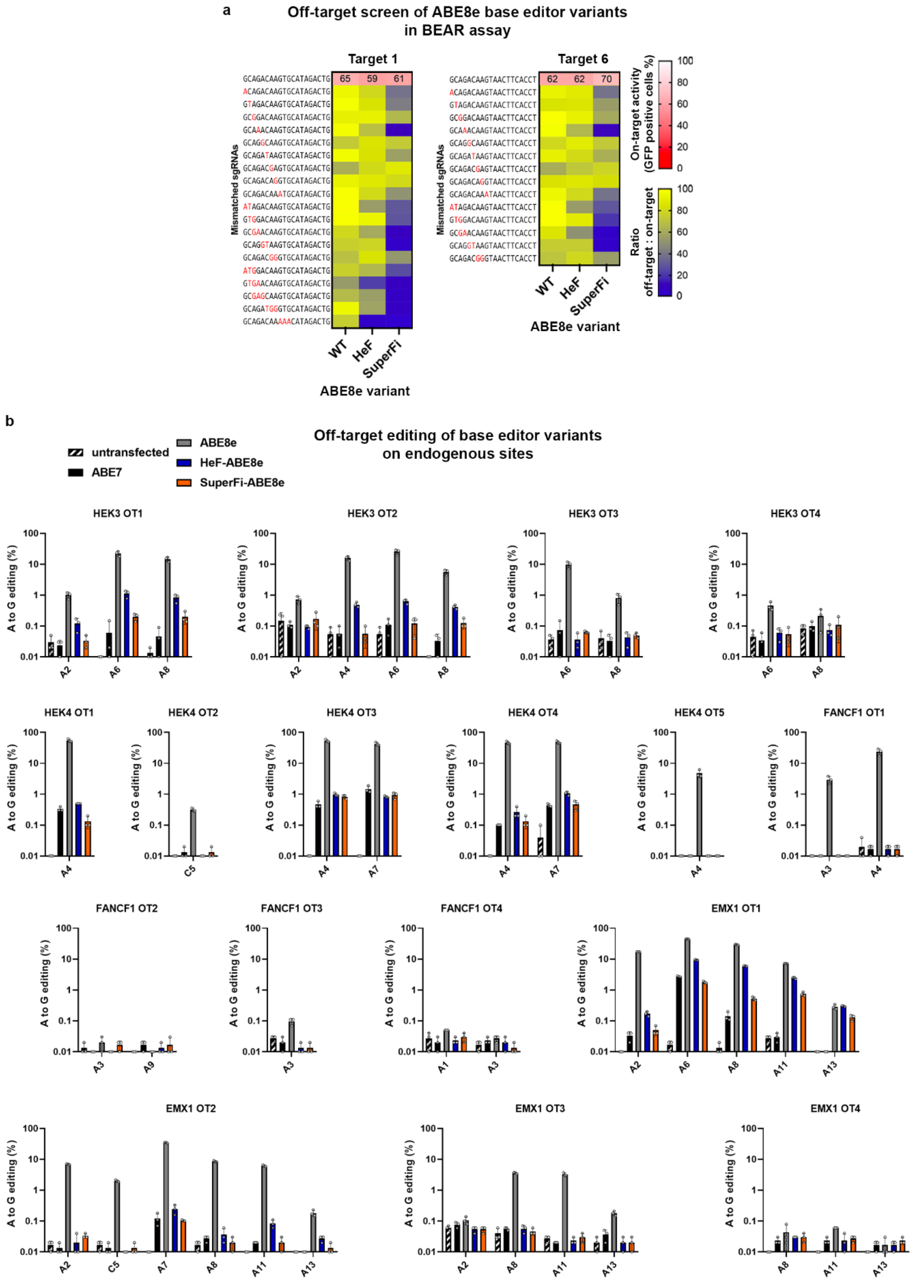


**Supplementary Figure 9. Off-target activities of SuperFi base editor variants in the BEAR assay and on endogenous target sites**

**a,** Mismatch tolerance of WT-ABE8e and IFN base editor variants as indicated in the panel were examined using 35 sgRNAs on two targets (Target 1 and 6 from Supplementary Figure 7a) with perfectly matching sgRNAs or sgRNAs mismatching in one, two or three positions as indicated by red letters in the spacer sequences. White

and red heatmaps show the mean on-target activity (as GFP positive cells) derived from triplicates. Blue and yellow heatmaps show the ratio of off-target/on-target activity derived from triplicates. **b,** Editing efficiencies of ABE7, WT-ABE8e, and two IFN ABE8e base editor variants are shown on 17 loci for assessing off-target base editing as measured by NGS. The diagrams show A to G (and in two cases C to G) conversion ratios at each adenine position for each target. As negative control, base conversion for non-transfected cells was measured (black with white dashes). Means are shown, error bars represent the standard deviation (SD) for triplicates (overlaid as white circles). **a-b,** Target sequences, BEAR assay and NGS data are reported in Supplementary Data files 1–3.

## Supplementary Figure 10

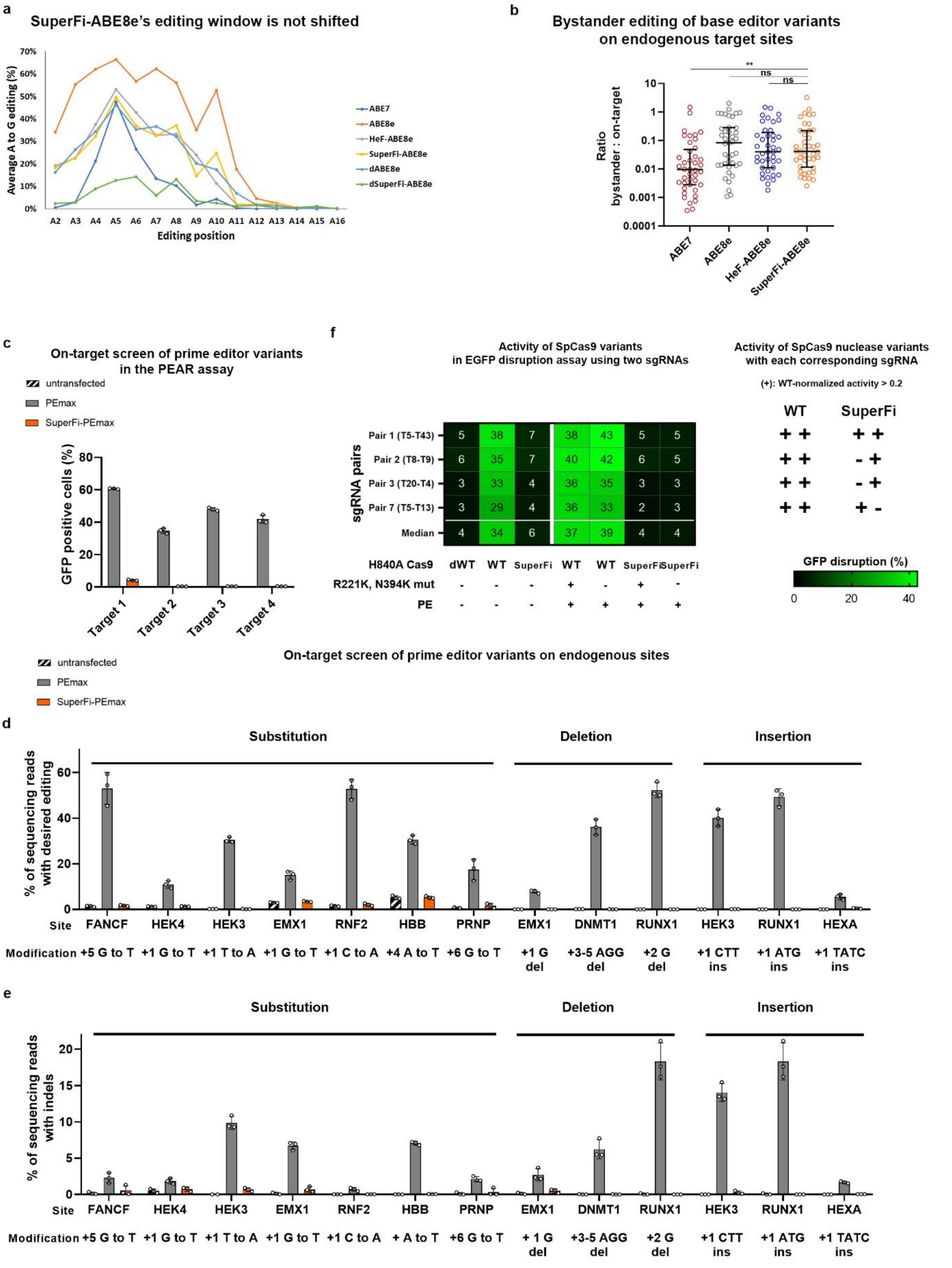


**Supplementary Figure 10. Activities of ABE base editors in different base positions and the activity of the SuperFi prime editor variant**

**a,** The editing window of the SuperFi ABE8e base editor is not shifted in comparison to ABE7. Data analysed here are from Supplementary Figure 7b, extended with dead ABE8e and dead SuperFi-ABE8e data. (*FANCF*s1, *EMX*1

and HEK4 data were excluded because no dead ABE8e and dead SuperFi-ABE8e data were available for those target sites.) Each point represents the average for that base position. **b,** Relative base editing activities (bystander/on-target ratio) of ABE variants are shown for all adenines (n=41) of 15 genomic sites (HEK4 does not have any bystander sites). The on-target value refers to the adenine base in the editing window with the highest editing, using ABE7 in case of each target site (except for EMX1 site, where ABE7 edit was close to zero on every position, thus we selected position A8, and in case of the RUNX site we selected position A3). Data are related to Supplementary Figure 7a. The results are presented on a scatter dot plot, the median and interquartile ranges are shown; data points are plotted as open circles representing the calculated values. Statistical significance was assessed by RM one-way ANOVA, statistical details and p-values are available in Methods and in Supplementary Data file 6 (ns: not significant, **p<0.01). **c,** Prime editing efficiencies of PEmax and SuperFi-PEmax, as indicated in the panel, were assessed in the PEAR fluorescent assay exploiting a pegRNA-target plasmid (PEAR-GFP-2in1)[33] bearing four different spacer and target sequences. **d**, Prime editing efficiencies (desired editing without indels) and **e**, unwanted indels of PEmax and SuperFi-PEmax were assessed by NGS on 10 genomic loci by installing 13 edits, including substitutions, insertions and deletions. As negative controls, base conversions are also shown for untransfected cells (black with white dashes). **c,d,e**, Means are shown, error bars represent the standard deviation (SD) for triplicates (overlaid as white circles). **f,** (left) On-target disruption activities of nickase SpCas9 variants with paired sgRNAs. The heatmap shows the mean on-target modifications (indels) of three parallel transfections. (right) +/- indicate the activities of the nuclease variants with the corresponding sgRNAs. **a-f,** Target sequences, PEAR assay, EGFP disruption data, NGS data and allele frequency tables are reported in Supplementary Data files 1–3 and 7.

**Supplementary Figure 11**

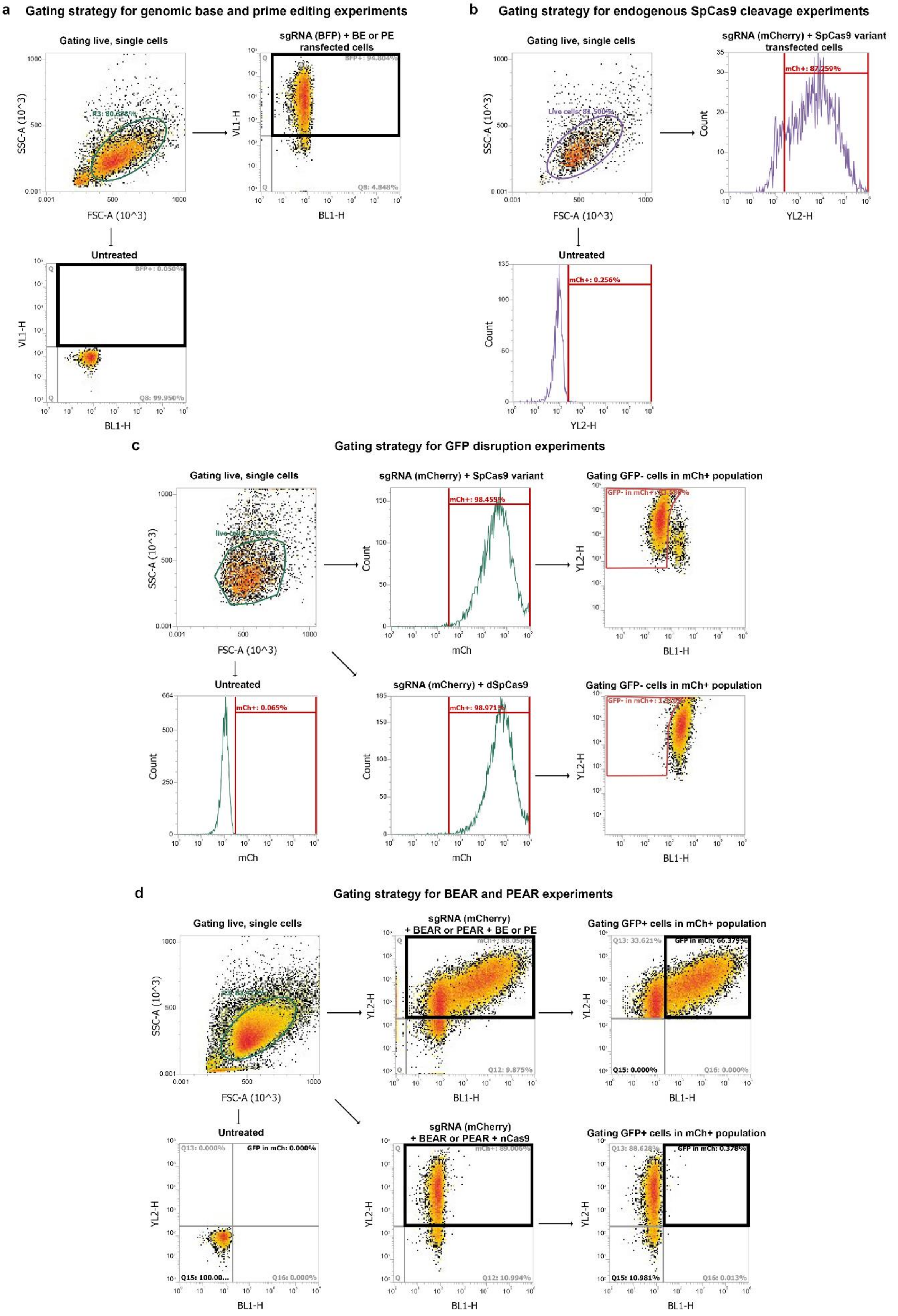


**Supplementary Figure 11. Flow cytometry gating examples are shown for experiments with N2a.dd-EGFP, N2a.EGFP, HEK293 and HEK293.EGFP cell lines**

**a-d,** Live, single cells were gated by FSC and SSC parameters (upper left panel). To establish mCherry or BFP negative gates untransfected cells were measured (bottom left panel). Cells were co-transfected with a sgRNA or pegRNA expressing plasmid bearing an mCherry or BFP expression cassette to follow transfection efficiency and a SpCas9 variant or a base or a prime editing construct. Transfected cells were gated (**a**) by BFP fluorescence excited by the 405 nm diode laser using the 440/50 filter for emission or (**b-d**) by mCherry fluorescence excited by the 561 nm diode laser using the 620/15 filter for emission (a, b: upper left panel; c, d: middle panels). **c, d,** In GFP disruption (in HEK-293.EGFP, N2a.dd-EGFP and N2a.-EGFP cell lines), BEAR or PEAR (in HEK293 cell line) experiments GFP reporter fluorescence (right panels) was determined by the 488 nm diode laser using the 530/30 filter for emission in the mCherry positive population. **c,** As a control, sgRNA expressing plasmid was co-transfected with a dead SpCas9 showing mCherry and baseline GFP fluorescence (bottom right panel). **d,** In case of BEAR or PEAR experiments a BEAR or PEAR reporter was also co-transfected hence GFP fluorescence could be detected in the mCherry positive population (upper right panel). As a control, sgRNA or pegRNA and the BEAR or PEAR plasmid was co-transfected with a nickase Cas9 showing mCherry and minimal background GFP fluorescence (bottom right panel).

# Supplementary Notes

For detailed primer, oligonucleotide, Addgene number and SpCas9 construct information see Supplementary Data file 1. The sequences of all plasmid constructs were confirmed by Sanger sequencing.

## Supplementary Note 1: Cas9 spacer cloning

sgRNA expression plasmids were constructed by ligating annealed DNA oligonucleotides harboring the spacer sequence with 4 nt-long overhangs into a *Bbs*I restriction enzyme digested pmCherry_gRNA (#80457), or pmCherry_gRNA_ver2 (Addgene #126776; this plasmid backbone lacks a truncated extra guideRNA scaffold sequence) plasmids[1]. A one-pot digestion-ligation protocol was followed[2]. The synthetic DNA oligonucleotides were hybridized and the annealed oligonucleotides (2.5 µM) with 50 ng plasmid, 3 units of *Bbs*I restriction enzyme and 1.5 units of T4 DNA ligase were mixed in Green buffer (Thermo Fisher Scientific) containing 500 µM ATP. The mixture was kept at 37 °C for one hour before transforming into chemically competent Stable Competent *E. coli* cells (NEB). Two single colonies, formed after culturing on an agar plate, were tested by restriction enzyme digestion and appropriate clones were sent for sequencing.

## Supplementary Note 2: SpCas9 variants, human expression plasmids

### pX330-Flag-WT SpCas9 (Addgene #126753)

All SpCas9 variant coding plasmids' backbone are identical with the hereunder shown sequence.

Whole DNA sequence of the plasmid:

Human codon optimized *S. pyogenes* Cas9 coloured in purple, NLS underlined, 3xFLAG tag in *light blue*, silent mutations underlined and marked with yellow.

CTAGAGGTACCCGTTACATAACTTACGGTAAATGGCCCGCCTGGCTGACCGCCCAACGACCCCCGCCCATTGAC
GTCAATAGTAACGCCAATAGGGACTTTCCATTGACGTCAATGGGTGGAGTATTTACGGTAAACTGCCCACTTG
GCAGTACATCAAGTGTATCATATGCCAAGTACGCCCCCTATTGACGTCAATGACGGTAAATGGCCCGCCTGGC
ATTGTGCCCAGTACATGACCTTATGGGACTTTCCTACTTGGCAGTACATCTACGTATTAGTCATCGCTATTACCA
TGGTCGAGGTGAGCCCCACGTTCTGCTTCACTCTCCCCATCTCCCCCCCCTCCCCACCCCCAATTTTGTATTTATT
TATTTTTTAATTATTTTGTGCAGCGATGGGGGCGGGGGGGGGGGGGGGGCGCGCCAGGCGGGGCGGGGC
GGGGCGAGGGGCGGGGCGGGGCGAGGCGGAGAGGTGCGGCGGCAGCCAATCAGAGCGGCGCGCTCCGAA

AGTTTCCTTTTATGGCGAGGCGGCGGCGGCGGCGGCCCTATAAAAAGCGAAGCGCGCGGCGGGCGGGAGTC

GCTGCGACGCTGCCTTCGCCCCGTGCCCCGCTCCGCCGCCGCCTCGCGCCGCCCGCCCCGGCTCTGACTGACCG

CGTTACTCCCACAGGTGAGCGGGCGGGACGGCCCTTCTCCTCCGGGCTGTAATTAGCTGAGCAAGAGGTAAG

GGTTTAAGGGATGGTTGGTTGGTGGGGTATTAATGTTTAATTACCTGGAGCACCTGCCTGAAATCACTTTTTTT

CAGGTTGGACCGGTGCCACCATG*GACTATAAGGACCACGACGGAGACTACAAGGATCATGATATTGATTACAA*

*AGACGATGACGATAAG*ATGGCCCCAAAGAAGAAGCGGAAGGTCGGTATCCACGGAGTCCCAGCAGCCGACA

AGAAGTACAGCATCGGCCTGGACATCGGCACCAACTCTGTGGGCTGGGCCGTGATCACCGACGAGTACAAGG

TGCCCAGCAAGAAATTCAAGGTGCTGGGCAACACCGACCGGCACAGCATCAAGAAGAACCTGATCGGAGCCC

TGCTGTTCGACAGCGGCGAAACAGCCGAGGCCACCCGGCTGAAGAGAACCGCCAGAAGAAGATACACCAGAC

GGAAGAACCGGATCTGCTATCTGCAAGAGATCTTCAGCAACGAGATGGCCAAGGTGGACGACAGCTTCTTCCA

CAGACTGGAAGAGTCCTTCCTGGTGGAAGAGGATAAGAAGCACGAGCGGCACCCCATCTTCGGCAACATCGT

GGACGAGGTGGCCTACCACGAGAAGTACCCCACCATCTACCACCTGAGAAAGAAACTGGTGGACAGCACCGA

CAAGGCCGACCTGCGGCTGATCTATCTGGCCCTGGCCCACATGATCAAGTTCCGGGGCCACTTCCTGATCGAG

GGCGACCTGAACCCCGACAACAGCGACGTGGACAAGCTGTTCATCCAGCTGGTGCAGACCTACAACCAGCTGT

TCGAGGAAAACCCCATCAACGCCAGCGGCGTGGACGCCAAGGCCATCCTGTCTGCCAGACTGAGCAAGAGCA

GACGGCTGGAAAATCTGATCGCCCAGCTGCCCGGCGAGAAGAAGAATGGCCTGTTCGGAAACCTGATTGCCC

TGAGCCTGGGCCTGACCCCCAACTTCAAGAGCAACTTCGACCTGGCCGAGGATGCCAAACTGCAGCTGAGCAA

GGACACCTACGACGACGACCTGGACAACCTGCTGGCCCAGATCGGCGACCAGTACGCCGACCTGTTTCTGGCC

GCCAAGAACCTGTCCGACGCCATCCTGCTGAGCGACATCCTGAGAGTGAACACCGAGATCACCAAGGCCCCCC

TGAGCGCCTCTATGATCAAGAGATACGACGAGCACCACCAGGACCTGACCCTGCTGAAAGCTCTCGTGCGGCA

GCAGCTGCCTGAGAAGTACAAAGAGATTTTCTTCGACCAGAGCAAGAACGGCTACGCCGGCTACATTGACGGC

GGAGCCAGCCAGGAAGAGTTCTACAAGTTCATCAAGCCCATCCTGGAAAAGATGGACGGCACCGAGGAACTG

CTCGTGAAGCTGAACAGAGAGGACCTGCTGCGGAAGCAGCGGACCTTCGACAACGGCAGCATCCCCCACCAG

ATCCACCTGGGAGAGCTGCACGCCATTCTGCGGCGGCAGGAAGATTTTTACCCATTCCTGAAGGACAACCGGG

AAAAGATCGAGAAGATCCTGACCTTCCGCATCCCCTACTACGTGGGCCCTCTGGCCAGGGGAAACAGCAGATT

CGCCTGGATGACCAGAAAGAGCGAGGAAACCATCACCCCCTGGAACTTCGAGGAAGTGGTGGACAAGGGCG

CTTCCGCCCAGAGCTTCATCGAGCGGATGACCAACTTCGATAAGAACCTGCCCAACGAGAAGGTGCTGCCCAA

GCACAGCCTGCTGTACGAGTACTTCACCGTGTATAACGAGCTGACCAAAGTGAAATACGTGACCGAGGGAATG

AGAAAGCCCGCCTTCCTGAGCGGCGAGCAGAAAAAGGCCATCGTGGACCTGCTGTTCAAGACCAACCGGAAA

GTGACCGTGAAGCAGCTGAAAGAGGACTACTTCAAGAAAATCGAGTGCTTCGACTCCGTGGAAATCTCCGGC

GTGGAAGATCGGTTCAACGCCTCCCTGGGCACATACCACGATCTGCTGAAAATTATCAAGGACAAGGACTTCC

TGGACAATGAGGAAAACGAGGACATTCTGGAAGATATCGTGCTGACCCTGACACTGTTTGAGGACAGAGAGA

TGATCGAGGAACGGCTGAAAACCTATGCCCACCTGTTCGACGACAAAGTGATGAAGCAGCTGAAGCGGCGGA

GATACACCGGCTGGGGCAGGCTGAGCCGGAAGCTGATCAACGGCATCCGGGACAAGCAGTCCGGCAAGACA

ATCCTGGATTTCCTGAAGTCCGACGGCTTCGCCAACAGAAACTTCATGCAGCTGATCCACGACGACAGCCTGAC

CTTTAAAGAGGACATCCAGAAAGCCCAGGTGTCCGGCCAGGGCGATAGCCTGCACGAGCACATTGCCAATCTG
GCCGGCAGCCCCGCCATTAAGAAGGGCATCCTGCAGACAGTGAAGGTGGTGGACGAGCTCGTGAAAGTGAT
GGGCCGGCACAAGCCCGAGAACATCGTGATCGAAATGGCCAGAGAGAACCAGACCACCCAGAAGGGACAGA
AGAACAGCCGCGAGAGAATGAAGCGGATCGAAGAGGGCATCAAAGAGCTGGGCAGCCAGATCCTGAAAGAA
CACCCCGTGGAAAACACCCAGCTGCAGAACGAGAAGCTGTACCTGTACTACCTGCAGAATGGGCGGGATATG
TACGTGGACCAGGAACTGGACATCAACCGGCTGTCCGACTACGATGTGGACCATATCGTGCCTCAGAGCTTTC
TGAAGGACGACTCCATCGACAACAAGGTGCTGACCAGAAGCGACAAGAACCGGGGCAAGAGCGACAACGTG
CCCTCCGAAGAGGTCGTGAAGAAGATGAAGAACTACTGGCGGCAGCTGCTGAACGCCAAGCTGATTACCCAG
AGAAAGTTCGACAATCTGACCAAGGCCGAGAGAGGCGGCCTGAGCGAACTGGATAAGGCCGGCTTCATCAAG
AGACAGCTGGTGGAAACCCGGCAGATCACAAAGCACGTGGCACAGATCCTGGACTCCCGGATGAACACTAAG
TACGACGAGAATGACAAGCTGATCCGGGAAGTGAAAGTGATCACCCTGAAGTCCAAGCTGGTGTCCGATTTCC
GGAAGGATTTCCAGTTTTACAAAGTGCGCGAGATCAACAACTACCACCACGCCCACGACGCGTACCTGAACGC
CGTCGTGGGAACCGCCCTGATCAAAAAGTACCCTAAGCTGGAAAGCGAGTTCGTGTACGGCGACTACAAGGT
GTACGACGTACGGAAGATGATCGCCAAGAGCGAGCAGGAAATCGGCAAGGCTACCGCCAAGTACTTCTTCTA
CAGCAACATCATGAACTTTTTCAAGACCGAGATTACCCTGGCCAACGGCGAGATCCGGAAGCGGCCTCTGATC
GAGACAAACGGCGAAACCGGGGAGATCGTGTGGGATAAGGGCCGGGATTTTGCCACCGTGCGGAAAGTGCT
GAGCATGCCCCAAGTGAATATCGTGAAAAAGACCGAGGTGCAGACAGGCGGCTTCAGCAAAGAGTCTATCCT
GCCCAAGAGGAACAGCGATAAGCTGATCGCCAGAAAGAAGGACTGGGACCCTAAGAAGTACGGCGGCTTCG
ACAGCCCCACCGTGGCCTATTCTGTGCTGGTGGTGGCCAAAGTGGAAAAGGGCAAGTCCAAGAAACTGAAGA
GTGTGAAAGAGCTGCTGGGGATCACCATCATGGAAAGAAGCAGCTTCGAGAAGAATCCCATCGACTTTCTGG
AAGCCAAGGGCTACAAAGAAGTGAAAAAGGACCTGATCATCAAGCTGCCTAAGTACTCCCTGTTCGAGCTGGA
AAACGGCCGGAAGAGAATGCTGGCCTCTGCCGGCGAACTGCAGAAGGGAAACGAACTGGCCCTGCCCTCCAA
ATATGTGAACTTCCTGTACCTGGCCAGCCACTATGAGAAGCTGAAGGGCTCCCCCGAGGATAATGAGCAGAAA
CAGCTGTTTGTGGAACAGCACAAGCACTACCTGGACGAGATCATCGAGCAGATCAGCGAGTTCTCCAAGAGA
GTGATCCTGGCCGACGCTAATCTGGACAAAGTGCTGTCCGCCTACAACAAGCACCGGGATAAGCCCATCAGAG
AGCAGGCCGAGAATATCATCCACCTGTTTACCCTGACCAATCTGGGAGCCCCTGCCGCCTTCAAGTACTTTGAC
ACCACCATCGACCGGAAGAGGTACACCAGCACCAAAGAGGTGCTGGACGCCACCCTGATCCACCAGAGCATC
ACCGGCCTGTACGAGACACGGATCGACCTGTCTCAGCTGGGAGGCGACAAAAGGCCGGCGGCCACGAAAAA
GGCCGGCCAGGCAAAAAAGAAAAAGTAAGAATTCCTAGAGCTCGCTGATCAGCCTCGACTGTGCCTTCTAGTT
GCCAGCCATCTGTTGTTTGCCCCTCCCCCGTGCCTTCCTTGACCCTGGAAGGTGCCACTCCCACTGTCCTTTCCT
AATAAAATGAGGAAATTGCATCGCATTGTCTGAGTAGGTGTCATTCTATTCTGGGGGGTGGGGTGGGGCAGG
ACAGCAAGGGGGAGGATTGGGAAGAGAATAGCAGGCATGCTGGGGAGCGGCCGCAGGAACCCCTAGTGAT
GGAGTTGGCCACTCCCTCTCTGCGCGCTCGCTCGCTCACTGAGGCCGGGCGACCAAAGGTCGCCCGACGCCCG
GGCTTTGCCCGGGCGGCCTCAGTGAGCGAGCGAGCGCGCAGCTGCCTGCAGGGGCGCCTGATGCGGTATTTT
CTCCTTACGCATCTGTGCGGTATTTCACACCGCATACGTCAAAGCAACCATAGTACGCGCCCTGTAGCGGCGCA

TTAAGCGCGGCGGGTGTGGTGGTTACGCGCAGCGTGACCGCTACACTTGCCAGCGCCCTAGCGCCCGCTCCTT
TCGCTTTCTTCCCTTCCTTTCTCGCCACGTTCGCCGGCTTTCCCCGTCAAGCTCTAAATCGGGGGCTCCCTTTAGG
GTTCCGATTTAGTGCTTTACGGCACCTCGACCCCAAAAAACTTGATTTGGGTGATGGTTCACGTAGTGGGCCAT
CGCCCTGATAGACGGTTTTTCGCCCTTTGACGTTGGAGTCCACGTTCTTTAATAGTGGACTCTTGTTCCAAACTG
GAACAACACTCAACCCTATCTCGGGCTATTCTTTTGATTTATAAGGGATTTTGCCGATTTCGGCCTATTGGTTAA
AAAATGAGCTGATTTAACAAAAATTTAACGCGAATTTTAACAAAATATTAACGTTTACAATTTTATGGTGCACTC
TCAGTACAATCTGCTCTGATGCCGCATAGTTAAGCCAGCCCCGACACCCGCCAACACCCGCTGACGCGCCCTGA
CGGGCTTGTCTGCTCCCGGCATCCGCTTACAGACAAGCTGTGACCGTCTCCGGGAGCTGCATGTGTCAGAGGT
TTTCACCGTCATCACCGAAACGCGCGAGACGAAAGGGCCTCGTGATACGCCTATTTTTATAGGTTAATGTCATG
ATAATAATGGTTTCTTAGACGTCAGGTGGCACTTTTCGGGGAAATGTGCGCGGAACCCCTATTTGTTTATTTTTC
TAAATACATTCAAATATGTATCCGCTCATGAGACAATAACCCTGATAAATGCTTCAATAATATTGAAAAAGGAA
GAGTATGAGTATTCAACATTTCCGTGTCGCCCTTATTCCCTTTTTTGCGGCATTTTGCCTTCCTGTTTTTGCTCAC
CCAGAAACGCTGGTGAAAGTAAAAGATGCTGAAGATCAGTTGGGTGCACGAGTGGGTTACATCGAACTGGAT
CTCAACAGCGGTAAGATCCTTGAGAGTTTTCGCCCCGAAGAACGTTTTCCAATGATGAGCACTTTTAAAGTTCT
GCTATGTGGCGCGGTATTATCCCGTATTGACGCCGGGCAAGAGCAACTCGGTCGCCGCATACACTATTCTCAG
AATGACTTGGTTGAGTACTCACCAGTCACAGAAAAGCATCTTACGGATGGCATGACAGTAAGAGAATTATGCA
GTGCTGCCATAACCATGAGTGATAACACTGCGGCCAACTTACTTCTGACAACGATCGGAGGACCGAAGGAGCT
AACCGCTTTTTTGCACAACATGGGGGATCATGTAACTCGCCTTGATCGTTGGGAACCGGAGCTGAATGAAGCC
ATACCAAACGACGAGCGTGACACCACGATGCCTGTAGCAATGGCAACAACGTTGCGCAAACTATTAACTGGCG
AACTACTTACTCTAGCTTCCCGGCAACAATTAATAGACTGGATGGAGGCGGATAAAGTTGCAGGACCACTTCT
GCGCTCGGCCCTTCCGGCTGGCTGGTTTATTGCTGATAAATCTGGAGCCGGTGAGCGTGGAAGCCGCGGTATC
ATTGCAGCACTGGGGCCAGATGGTAAGCCCTCCCGTATCGTAGTTATCTACACGACGGGGAGTCAGGCAACTA
TGGATGAACGAAATAGACAGATCGCTGAGATAGGTGCCTCACTGATTAAGCATTGGTAACTGTCAGACCAAGT
TTACTCATATATACTTTAGATTGATTTAAAACTTCATTTTTAATTTAAAAGGATCTAGGTGAAGATCCTTTTTGAT
AATCTCATGACCAAAATCCCTTAACGTGAGTTTTCGTTCCACTGAGCGTCAGACCCCGTAGAAAAGATCAAAGG
ATCTTCTTGAGATCCTTTTTTTCTGCGCGTAATCTGCTGCTTGCAAACAAAAAAACCACCGCTACCAGCGGTGGT
TTGTTTGCCGGATCAAGAGCTACCAACTCTTTTTCCGAAGGTAACTGGCTTCAGCAGAGCGCAGATACCAAATA
CTGTCCTTCTAGTGTAGCCGTAGTTAGGCCACCACTTCAAGAACTCTGTAGCACCGCCTACATACCTCGCTCTGC
TAATCCTGTTACCAGTGGCTGCTGCCAGTGGCGATAAGTCGTGTCTTACCGGGTTGGACTCAAGACGATAGTT
ACCGGATAAGGCGCAGCGGTCGGGCTGAACGGGGGGTTCGTGCACACAGCCCAGCTTGGAGCGAACGACCT
ACACCGAACTGAGATACCTACAGCGTGAGCTATGAGAAAGCGCCACGCTTCCCGAAGGGAGAAAGGCGGACA
GGTATCCGGTAAGCGGCAGGGTCGGAACAGGAGAGCGCACGAGGGAGCTTCCAGGGGGAAACGCCTGGTAT
CTTTATAGTCCTGTCGGGTTTCGCCACCTCTGACTTGAGCGTCGATTTTTGTGATGCTCGTCAGGGGGGCGGAG
CCTATGGAAAAACGCCAGCAACGCGGCCTTTTTACGGTTCCTGGCCTTTTGCTGGCCTTTTGCTCACATG

## pX330-Flag-SpCas9-HF1 (Addgene #126755)

DNA sequence of the SpCas9-HF1 coding cassette:

Human codon optimized *S. pyogenes* Cas9 coloured in purple, NLS underlined, 3xFLAG tag in *light blue*, silent mutations underlined and marked with yellow.

ATG*GACTATAAGGACCACGACGGAGACTACAAGGATCATGATATTGATTACAAAGACGATGACGATAAG*ATG
GCCCCAAAGAAGAAGCGGAAGGTCGGTATCCACGGAGTCCCAGCAGCCGACAAGAAGTACAGCATCGGCCTG
GACATCGGCACCAACTCTGTGGGCTGGGCCGTGATCACCGACGAGTACAAGGTGCCCAGCAAGAAATTCAAG
GTGCTGGGCAACACCGACCGGCACAGCATCAAGAAGAACCTGATCGGAGCCCTGCTGTTCGACAGCGGCGAA
ACAGCCGAGGCCACCCGGCTGAAGAGAACCGCCAGAAGAAGATACACCAGACGGAAGAACCGGATCTGCTAT
CTGCAAGAGATCTTCAGCAACGAGATGGCCAAGGTGGACGACAGCTTCTTCCACAGACTGGAAGAGTCCTTCC
TGGTGGAAGAGGATAAGAAGCACGAGCGGCACCCCATCTTCGGCAACATCGTGGACGAGGTGGCCTACCACG
AGAAGTACCCCACCATCTACCACCTGAGAAAGAAACTGGTGGACAGCACCGACAAGGCCGACCTGCGGCTGA
TCTATCTGGCCCTGGCCCACATGATCAAGTTCCGGGGCCACTTCCTGATCGAGGGCGACCTGAACCCCGACAA
CAGCGACGTGGACAAGCTGTTCATCCAGCTGGTGCAGACCTACAACCAGCTGTTCGAGGAAAACCCCATCAAC
GCCAGCGGCGTGGACGCCAAGGCCATCCTGTCTGCCAGACTGAGCAAGAGCAGACGGCTGGAAAATCTGATC
GCCCAGCTGCCCGGCGAGAAGAAGAATGGCCTGTTCGGAAACCTGATTGCCCTGAGCCTGGGCCTGACCCCC
AACTTCAAGAGCAACTTCGACCTGGCCGAGGATGCCAAACTGCAGCTGAGCAAGGACACCTACGACGACGAC
CTGGACAACCTGCTGGCCCAGATCGGCGACCAGTACGCCGACCTGTTTCTGGCCGCCAAGAACCTGTCCGACG
CCATCCTGCTGAGCGACATCCTGAGAGTGAACACCGAGATCACCAAGGCCCCCCTGAGCGCCTCTATGATCAA
GAGATACGACGAGCACCACCAGGACCTGACCCTGCTGAAAGCTCTCGTGCGGCAGCAGCTGCCTGAGAAGTA
CAAAGAGATTTTCTTCGACCAGAGCAAGAACGGCTACGCCGGCTACATTGACGGCGGAGCCAGCCAGGAAGA
GTTCTACAAGTTCATCAAGCCCATCCTGGAAAAGATGGACGGCACCGAGGAACTGCTCGTGAAGCTGAACAGA
GAGGACCTGCTGCGGAAGCAGCGGACCTTCGACAACGGCAGCATCCCCCACCAGATCCACCTGGGAGAGCTG
CACGCCATTCTGCGGCGGCAGGAAGATTTTTACCCATTCCTGAAGGACAACCGGGAAAAGATCGAGAAGATCC
TGACCTTCCGCATCCCCTACTACGTGGGCCCTCTGGCCAGGGGAAACAGCAGATTCGCCTGGATGACCAGAAA
GAGCGAGGAAACCATCACCCCCTGGAACTTCGAGGAAGTGGTGGACAAGGGCGCTTCCGCCCAGAGCTTCAT
CGAGCGGATGACCGCCTTCGATAAGAACCTGCCCAACGAGAAGGTGCTGCCCAAGCACAGCCTGCTGTACGA
GTACTTCACCGTGTATAACGAGCTGACCAAAGTGAAATACGTGACCGAGGGAATGAGAAAGCCCGCCTTCCTG
AGCGGCGAGCAGAAAAAGGCCATCGTGGACCTGCTGTTCAAGACCAACCGGAAAGTGACCGTGAAGCAGCTG
AAAGAGGACTACTTCAAGAAAATCGAGTGCTTCGACTCCGTGGAAATCTCCGGCGTGGAAGATCGGTTCAACG
CCTCCCTGGGCACATACCACGATCTGCTGAAAATTATCAAGGACAAGGACTTCCTGGACAATGAGGAAAACGA
GGACATTCTGGAAGATATCGTGCTGACCCTGACACTGTTTGAGGACAGAGAGATGATCGAGGAACGGCTGAA

AACCTATGCCCACCTGTTCGACGACAAAGTGATGAAGCAGCTGAAGCGGCGGAGATACACCGGCTGGGGCGC
GCTGAGCCGGAAGCTGATCAACGGCATCCGCGACAAGCAGTCCGGCAAGACAATCCTGGATTTCCTGAAGTCC
GACGGCTTCGCCAACAGAAACTTCATGGCGCTGATCCACGACGACAGCCTGACCTTTAAAGAGGACATCCAGA
AAGCCCAGGTGTCCGGCCAGGGCGATAGCCTGCACGAGCACATTGCCAATCTCGCCGGCAGCCCCGCCATTAA
GAAGGGCATCCTCCAGACAGTGAAGGTGGTGGACGAGCTGGTGAAAGTGATGGGCCGGCACAAGCCCGAGA
ACATCGTGATCGAAATGGCCAGAGAGAACCAGACCACCCAGAAGGGACAGAAGAACAGTCGCGAGAGAATG
AAGCGGATCGAAGAGGGCATCAAAGAGCTGGGCAGCCAGATCCTGAAAGAACACCCCGTGGAAAACACCCA
GCTCCAGAACGAGAAGCTGTACCTGTACTACCTCCAGAATGGGCGGGATATGTACGTGGACCAGGAACTGGA
CATCAACCGGCTGTCCGACTACGATGTGGACCATATCGTGCCTCAGAGCTTTCTTAAGGACGACTCCATCGACA
ACAAGGTGCTGACCAGAAGCGACAAGAACCGGGGCAAGAGCGACAACGTGCCCTCCGAAGAGGTCGTGAAG
AAGATGAAGAACTACTGGCGGCAGCTGCTGAACGCCAAGCTGATTACCCAGAGAAAGTTCGACAATCTGACC
AAGGCCGAGAGAGGCGGCCTGAGCGAACTGGATAAGGCCGGCTTCATCAAGAGACAGCTGGTGGAAACCCG
GGCGATCACAAAGCACGTGGCACAGATCCTGGACTCCCGGATGAACACTAAGTACGACGAGAATGACAAGCT
GATCCGGGAAGTGAAAGTGATCACCCTGAAGTCCAAGCTGGTGTCCGATTTCCGGAAGGATTTCCAGTTTTAC
AAAGTGCGCGAGATCAACAACTACCACCACGCCCACGACGCGTACCTGAACGCCGTCGTGGGAACCGCCCTGA
TCAAAAAGTACCCTAAGCTGGAAAGCGAGTTCGTGTACGGCGACTACAAGGTGTACGACGTACGGAAGATGA
TCGCCAAGAGCGAGCAGGAAATCGGCAAGGCTACCGCCAAGTACTTCTTCTACAGCAACATCATGAACTTTTTC
AAGACCGAGATTACCCTGGCCAACGGCGAGATCCGGAAGCGGCCTCTGATCGAGACAAACGGCGAAACCGG
GGAGATCGTGTGGGATAAGGGCCGGGATTTTGCCACCGTGCGGAAAGTGCTGAGCATGCCCCAAGTGAATAT
CGTGAAAAAGACCGAGGTGCAGACAGGCGGCTTCAGCAAAGAGTCTATCCTGCCCAAGAGGAACAGCGATAA
GCTGATCGCCAGAAAGAAGGACTGGGATCCTAAGAAGTACGGCGGCTTCGACAGCCCCACCGTGGCCTATTCT
GTGCTGGTGGTGGCCAAAGTGGAAAAGGGCAAGTCCAAGAAGCTTAAGAGTGTGAAAGAGCTGCTGGGGAT
CACCATCATGGAAAGAAGCAGCTTCGAGAAGAATCCCATCGACTTTCTGGAAGCCAAGGGCTACAAAGAAGT
GAAAAAGGACCTGATCATCAAGCTGCCTAAGTACTCGCTCTTCGAGCTGGAAAACGGCCGGAAGAGAATGCT
GGCCTCTGCCGGCGAACTGCAGAAGGGAAACGAACTGGCCCTGCCCTCCAAATATGTGAACTTCCTGTACCTG
GCCAGCCACTATGAGAAGCTGAAGGGCTCCCCCGAGGATAATGAGCAGAAACAGCTGTTTGTGGAACAGCAC
AAGCACTACCTGGACGAGATCATCGAGCAGATCAGCGAGTTCTCCAAGAGAGTGATCCTGGCCGACGCTAATC
TGGACAAAGTGCTGTCCGCCTACAACAAGCACCGGGATAAGCCCATCAGAGAGCAGGCCGAGAATATCATCC
ACCTGTTTACCCTGACCAATCTGGGAGCCCCTGCCGCCTTCAAGTACTTTGACACCACCATCGACCGGAAGAGG
TACACCAGCACCAAAGAGGTGCTGGACGCCACCCTGATCCACCAGAGCATCACCGGCCTGTACGAGACACGG
ATCGACCTGTCTCAGCTGGGAGGCGACAAAAGGCCGGCGGCCACGAAAAAGGCCGGCCAGGCAAAAAAGAA
AAAGTAA

# pX330-Flag-HypaSpCas9 (Addgene #126756)

DNA sequence of the HypaSpCas9 coding cassette:

Human codon optimized *S. pyogenes* Cas9 coloured in purple, NLS underlined, 3xFLAG tag in *light blue*, silent mutations underlined and marked with yellow.

ATG*GACTATAAGGACCACGACGGAGACTACAAGGATCATGATATTGATTACAAAGACGATGACGATAAG*ATG
GCCCCAAAGAAGAAGCGGAAGGTCGGTATCCACGGAGTCCCAGCAGCCGACAAGAAGTACAGCATCGGCCTG
GACATCGGCACCAACTCTGTGGGCTGGGCCGTGATCACCGACGAGTACAAGGTGCCCAGCAAGAAATTCAAG
GTGCTGGGCAACACCGACCGGCACAGCATCAAGAAGAACCTGATCGGAGCCCTGCTGTTCGACAGCGGCGAA
ACAGCCGAGGCCACCCGGCTGAAGAGAACCGCCAGAAGAAGATACACCAGACGGAAGAACCGGATCTGCTAT
CTGCAAGAGATCTTCAGCAACGAGATGGCCAAGGTGGACGACAGCTTCTTCCACAGACTGGAAGAGTCCTTCC
TGGTGGAAGAGGATAAGAAGCACGAGCGGCACCCCATCTTCGGCAACATCGTGGACGAGGTGGCCTACCACG
AGAAGTACCCCACCATCTACCACCTGAGAAAGAAACTGGTGGACAGCACCGACAAGGCCGACCTGCGGCTGA
TCTATCTGGCCCTGGCCCACATGATCAAGTTCCGGGGCCACTTCCTGATCGAGGGCGACCTGAACCCCGACAA
CAGCGACGTGGACAAGCTGTTCATCCAGCTGGTGCAGACCTACAACCAGCTGTTCGAGGAAAACCCCATCAAC
GCCAGCGGCGTGGACGCCAAGGCCATCCTGTCTGCCAGACTGAGCAAGAGCAGACGGCTGGAAAATCTGATC
GCCCAGCTGCCCGGCGAGAAGAAGAATGGCCTGTTCGGAAACCTGATTGCCCTGAGCCTGGGCCTGACCCCC
AACTTCAAGAGCAACTTCGACCTGGCCGAGGATGCCAAACTGCAGCTGAGCAAGGACACCTACGACGACGAC
CTGGACAACCTGCTGGCCCAGATCGGCGACCAGTACGCCGACCTGTTTCTGGCCGCCAAGAACCTGTCCGACG
CCATCCTGCTGAGCGACATCCTGAGAGTGAACACCGAGATCACCAAGGCCCCCCTGAGCGCCTCTATGATCAA
GAGATACGACGAGCACCACCAGGACCTGACCCTGCTGAAAGCTCTCGTGCGGCAGCAGCTGCCTGAGAAGTA
CAAAGAGATTTTCTTCGACCAGAGCAAGAACGGCTACGCCGGCTACATTGACGGCGGAGCCAGCCAGGAAGA
GTTCTACAAGTTCATCAAGCCCATCCTGGAAAAGATGGACGGCACCGAGGAACTGCTCGTGAAGCTGAACAGA
GAGGACCTGCTGCGGAAGCAGCGGACCTTCGACAACGGCAGCATCCCCCACCAGATCCACCTGGGAGAGCTG
CACGCCATTCTGCGGCGGCAGGAAGATTTTTACCCATTCCTGAAGGACAACCGGGAAAAGATCGAGAAGATCC
TGACCTTCCGCATCCCCTACTACGTGGGCCCTCTGGCCAGGGGAAACAGCAGATTCGCCTGGATGACCAGAAA
GAGCGAGGAAACCATCACCCCCTGGAACTTCGAGGAAGTGGTGGACAAGGGCGCTTCCGCCCAGAGCTTCAT
CGAGCGGATGACCAACTTCGATAAGAACCTGCCCAACGAGAAGGTGCTGCCCAAGCACAGCCTGCTGTACGA
GTACTTCACCGTGTATAACGAGCTGACCAAAGTGAAATACGTGACCGAGGGAATGAGAAAGCCCGCCTTCCTG
AGCGGCGAGCAGAAAAAGGCCATCGTGGACCTGCTGTTCAAGACCAACCGGAAAGTGACCGTGAAGCAGCTG
AAAGAGGACTACTTCAAGAAAATCGAGTGCTTCGACTCCGTGGAAATCTCCGGCGTGGAAGATCGGTTCAACG
CCTCCCTGGGCACATACCACGATCTGCTGAAAATTATCAAGGACAAGGACTTCCTGGACAATGAGGAAAACGA
GGACATTCTGGAAGATATCGTGCTGACCCTGACACTGTTTGAGGACAGAGAGATGATCGAGGAACGGCTGAA

AACCTATGCCCACCTGTTCGACGACAAAGTGATGAAGCAGCTGAAGCGGCGGAGATACACCGGCTGGGGCAG
GCTGAGCCGGAAGCTGATCAACGGCATCCGCGACAAGCAGTCCGGCAAGACAATCCTGGATTTCCTGAAGTCC
GACGGCTTCGCCAACAGAGCCTTTGCAGCCCTGATCGCTGACGACAGCCTGACCTTTAAAGAGGACATCCAGA
AAGCCCAGGTGTCCGGCCAGGGCGATAGCCTGCACGAGCACATTGCCAATCTGGCCGGCAGCCCCGCCATTA
AGAAGGGCATCCTGCAGACAGTGAAGGTGGTGGACGAGCTCGTGAAAGTGATGGGCCGGCACAAGCCCGAG
AACATCGTGATCGAAATGGCCAGAGAGAACCAGACCACCCAGAAGGGACAGAAGAACAGCCGCGAGAGAAT
GAAGCGGATCGAAGAGGGCATCAAAGAGCTGGGCAGCCAGATCCTGAAAGAACACCCCGTGGAAAACACCC
AGCTGCAGAACGAGAAGCTGTACCTGTACTACCTGCAGAATGGGCGGGATATGTACGTGGACCAGGAACTGG
ACATCAACCGGCTGTCCGACTACGATGTGGACCATATCGTGCCTCAGAGCTTTCTGAAGGACGACTCCATCGAC
AACAAGGTGCTGACCAGAAGCGACAAGAACCGGGGCAAGAGCGACAACGTGCCCTCCGAAGAGGTCGTGAA
GAAGATGAAGAACTACTGGCGGCAGCTGCTGAACGCCAAGCTGATTACCCAGAGAAAGTTCGACAATCTGAC
CAAGGCCGAGAGAGGCGGCCTGAGCGAACTGGATAAGGCCGGCTTCATCAAGAGACAGCTGGTGGAAACCC
GGCAGATCACAAAGCACGTGGCACAGATCCTGGACTCCCGGATGAACACTAAGTACGACGAGAATGACAAGC
TGATCCGGGAAGTGAAAGTGATCACCCTGAAGTCCAAGCTGGTGTCCGATTTCCGGAAGGATTTCCAGTTTTA
CAAAGTGCGCGAGATCAACAACTACCACCACGCCCACGACGCGTACCTGAACGCCGTCGTGGGAACCGCCCTG
ATCAAAAAGTACCCTAAGCTGGAAAGCGAGTTCGTGTACGGCGACTACAAGGTGTACGACGTACGGAAGATG
ATCGCCAAGAGCGAGCAGGAAATCGGCAAGGCTACCGCCAAGTACTTCTTCTACAGCAACATCATGAACTTTTT
CAAGACCGAGATTACCCTGGCCAACGGCGAGATCCGGAAGCGGCCTCTGATCGAGACAAACGGCGAAACCGG
GGAGATCGTGTGGGATAAGGGCCGGGATTTTGCCACCGTGCGGAAAGTGCTGAGCATGCCCCAAGTGAATAT
CGTGAAAAAGACCGAGGTGCAGACAGGCGGCTTCAGCAAAGAGTCTATCCTGCCCAAGAGGAACAGCGATAA
GCTGATCGCCAGAAAGAAGGACTGGGACCCTAAGAAGTACGGCGGCTTCGACAGCCCCACCGTGGCCTATTC
TGTGCTGGTGGTGGCCAAAGTGGAAAAGGGCAAGTCCAAGAAACTGAAGAGTGTGAAAGAGCTGCTGGGGA
TCACCATCATGGAAAGAAGCAGCTTCGAGAAGAATCCCATCGACTTTCTGGAAGCCAAGGGCTACAAAGAAGT
GAAAAAGGACCTGATCATCAAGCTGCCTAAGTACTCCCTGTTCGAGCTGGAAAACGGCCGGAAGAGAATGCT
GGCCTCTGCCGGCGAACTGCAGAAGGGAAACGAACTGGCCCTGCCCTCCAAATATGTGAACTTCCTGTACCTG
GCCAGCCACTATGAGAAGCTGAAGGGCTCCCCCGAGGATAATGAGCAGAAACAGCTGTTTGTGGAACAGCAC
AAGCACTACCTGGACGAGATCATCGAGCAGATCAGCGAGTTCTCCAAGAGAGTGATCCTGGCCGACGCTAATC
TGGACAAAGTGCTGTCCGCCTACAACAAGCACCGGGATAAGCCCATCAGAGAGCAGGCCGAGAATATCATCC
ACCTGTTTACCCTGACCAATCTGGGAGCCCCTGCCGCCTTCAAGTACTTTGACACCACCATCGACCGGAAGAGG
TACACCAGCACCAAAGAGGTGCTGGACGCCACCCTGATCCACCAGAGCATCACCGGCCTGTACGAGACACGG
ATCGACCTGTCTCAGCTGGGAGGCGACAAAAGGCCGGCGGCCACGAAAAAGGCCGGCCAGGCAAAAAAGAA
AAAGTAA

<u>pX330-Flag-evoSpCas9 (Addgene #126758)</u>

DNA sequence of the HypaSpCas9 coding cassette:

Human codon optimized *S. pyogenes* Cas9 coloured in purple, NLS underlined, 3xFLAG tag in *light blue*, silent mutations underlined and marked with <u>yellow</u>.

ATG*GACTATAAGGACCACGACGGAGACTACAAGGATCATGATATTGATTACAAAGACGATGACGATAAG*ATG
GCC<u>CCAAAGAAGAAGCGGAAGGTC</u>GGTATCCACGGAGTCCCAGCAGCCGACAAGAAGTACAGCATCGGCCTG
GACATCGGCACCAACTCTGTGGGCTGGGCCGTGATCACCGACGAGTACAAGGTGCCCAGCAAGAAATTCAAG
GTGCTGGGCAACACCGACCGGCACAGCATCAAGAAGAACCTGATCGGAGCCCTGCTGTTCGACAGCGGCGAA
ACAGCCGAGGCCACCCGGCTGAAGAGAACCGCCAGAAGAAGATACACCAGACGGAAGAACCGGATCTGCTAT
CTGCAAGAGATCTTCAGCAACGAGATGGCCAAGGTGGACGACAGCTTCTTCCACAGACTGGAAGAGTCCTTCC
TGGTGGAAGAGGATAAGAAGCACGAGCGGCACCCCATCTTCGGCAACATCGTGGACGAGGTGGCCTACCACG
AGAAGTACCCCACCATCTACCACCTGAGAAAGAAACTGGTGGACAGCACCGACAAGGCCGACCTGCGGCTGA
TCTATCTGGCCCTGGCCCACATGATCAAGTTCCGGGGCCACTTCCTGATCGAGGGCGACCTGAACCCCGACAA
CAGCGACGTGGACAAGCTGTTCATCCAGCTGGTGCAGACCTACAACCAGCTGTTCGAGGAAAACCCCATCAAC
GCCAGCGGCGTGGACGCCAAGGCCATCCTGTCTGCCAGACTGAGCAAGAGCAGACGGCTGGAAAATCTGATC
GCCCAGCTGCCCGGCGAGAAGAAGAATGGCCTGTTCGGAAACCTGATTGCCCTGAGCCTGGGCCTGACCCCC
AACTTCAAGAGCAACTTCGACCTGGCCGAGGATGCCAAACTGCAGCTGAGCAAGGACACCTACGACGACGAC
CTGGACAACCTGCTGGCCCAGATCGGCGACCAGTACGCCGACCTGTTTCTGGCCGCCAAGAACCTGTCCGACG
CCATCCTGCTGAGCGACATCCTGAGAGTGAACACCGAGATCACCAAGGCCCCCCTGAGCGCCTCTATGATCAA
GAGATACGACGAGCACCACCAGGACCTGACCCTGCTGAAAGCTCTCGTGCGGCAGCAGCTGCCTGAGAAGTA
CAAAGAGATTTTCTTCGACCAGAGCAAGAACGGCTACGCCGGCTACATTGACGGCGGAGCCAGCCAGGAAGA
GTTCTACAAGTTCATCAAGCCCATCCTGGAAAAGATGGACGGCACCGAGGAACTGCTCGTGAAGCTGAACAGA
GAGGACCTGCTGCGGAAGCAGCGGACCTTCGACAACGGCAGCATCCCCCACCAGATCCACCTGGGAGAGCTG
CACGCCATTCTGCGGCGGCAGGAAGATTTTTACCCATTCCTGAAGGACAACCGGGAAAAGATCGAGAAGATCC
TGACCTTCCGCATCCCCTACTACGTGGGCCCTCTGGCCAGGGGAAACAGCAGATTCGCCTGGATGACCAGAAA
GAGCGAGGAAACCATCACCCCCTGGAACTTCGAGGAAGTGGTGGACAAGGGCGCTTCCGCCCAGAGCTTCAT
CGAGCGGGTGACCAACTTCGATAAGAACCTGCCCAACGAGAAGGTGCTGCCCAAGCACAGCCTGCTGAACGA
GTACTTCACCGTGTATAACGAGCTGACCGAAGTGAAATACGTGACCGAGGGAATGAGAAAGCCCGCCTTCCTG
AGCGGCGAGCAGAAAAAGGCCATCGTGGACCTGCTGTTCAAGACCAACCGGAAAGTGACCGTGAAGCAGCTG
AAAGAGGACTACTTCAAGAAAATCGAGTGCTTCGACTCCGTGGAAATCTCCGGCGTGGAAGATCGGTTCAACG
CCTCCCTGGGCACATACCACGATCTGCTGAAAATTATCAAGGACAAGGACTTCCTGGACAATGAGGAAAACGA
GGACATTCTGGAAGATATCGTGCTGACCCTGACACTGTTTGAGGACAGAGAGATGATCGAGGAACGGCTGAA

AACCTATGCCCACCTGTTCGACGACAAAGTGATGAAGCAGCTGAAGCGGCGGAGATACACCGGCTGGGGCCA
GCTGAGCCGGAAGCTGATCAACGGCATCCGGGACAAGCAGTCCGGCAAGACAATCCTGGATTTCCTGAAGTC
CGACGGCTTCGCCAACAGAAACTTCATGCAGCTGATCCACGACGACAGCCTGACCTTTAAAGAGGACATCCAG
AAAGCCCAGGTGTCCGGCCAGGGCGATAGCCTGCACGAGCACATTGCCAATCTGGCCGGCAGCCCCGCCATT
AAGAAGGGCATCCTGCAGACAGTGAAGGTGGTGGACGAGCTCGTGAAAGTGATGGGCCGGCACAAGCCCGA
GAACATCGTGATCGAAATGGCCAGAGAGAACCAGACCACCCAGAAGGGACAGAAGAACAGCCGCGAGAGAA
TGAAGCGGATCGAAGAGGGCATCAAAGAGCTGGGCAGCCAGATCCTGAAAGAACACCCCGTGGAAAACACC
CAGCTGCAGAACGAGAAGCTGTACCTGTACTACCTGCAGAATGGGCGGGATATGTACGTGGACCAGGAACTG
GACATCAACCGGCTGTCCGACTACGATGTGGACCATATCGTGCCTCAGAGCTTTCTGAAGGACGACTCCATCG
ACAACAAGGTGCTGACCAGAAGCGACAAGAACCGGGGCAAGAGCGACAACGTGCCCTCCGAAGAGGTCGTG
AAGAAGATGAAGAACTACTGGCGGCAGCTGCTGAACGCCAAGCTGATTACCCAGAGAAAGTTCGACAATCTG
ACCAAGGCCGAGAGAGGCGGCCTGAGCGAACTGGATAAGGCCGGCTTCATCAAGAGACAGCTGGTGGAAAC
CCGGCAGATCACAAAGCACGTGGCACAGATCCTGGACTCCCGGATGAACACTAAGTACGACGAGAATGACAA
GCTGATCCGGGAAGTGAAAGTGATCACCCTGAAGTCCAAGCTGGTGTCCGATTTCCGGAAGGATTTCCAGTTT
TACAAAGTGCGCGAGATCAACAACTACCACCACGCCCACGACGCGTACCTGAACGCCGTCGTGGGAACCGCCC
TGATCAAAAAGTACCCTAAGCTGGAAAGCGAGTTCGTGTACGGCGACTACAAGGTGTACGACGTACGGAAGA
TGATCGCCAAGAGCGAGCAGGAAATCGGCAAGGCTACCGCCAAGTACTTCTTCTACAGCAACATCATGAACTT
TTTCAAGACCGAGATTACCCTGGCCAACGGCGAGATCCGGAAGCGGCCTCTGATCGAGACAAACGGCGAAAC
CGGGGAGATCGTGTGGGATAAGGGCCGGGATTTTGCCACCGTGCGGAAAGTGCTGAGCATGCCCCAAGTGA
ATATCGTGAAAAAGACCGAGGTGCAGACAGGCGGCTTCAGCAAAGAGTCTATCCTGCCCAAGAGGAACAGCG
ATAAGCTGATCGCCAGAAAGAAGGACTGGGACCCTAAGAAGTACGGCGGCTTCGACAGCCCCACCGTGGCCT
ATTCTGTGCTGGTGGTGGCCAAAGTGGAAAAGGGCAAGTCCAAGAAACTGAAGAGTGTGAAAGAGCTGCTG
GGGATCACCATCATGGAAAGAAGCAGCTTCGAGAAGAATCCCATCGACTTTCTGGAAGCCAAGGGCTACAAA
GAAGTGAAAAAGGACCTGATCATCAAGCTGCCTAAGTACTCCCTGTTCGAGCTGGAAAACGGCCGGAAGAGA
ATGCTGGCCTCTGCCGGCGAACTGCAGAAGGGAAACGAACTGGCCCTGCCCTCCAAATATGTGAACTTCCTGT
ACCTGGCCAGCCACTATGAGAAGCTGAAGGGCTCCCCCGAGGATAATGAGCAGAAACAGCTGTTTGTGGAAC
AGCACAAGCACTACCTGGACGAGATCATCGAGCAGATCAGCGAGTTCTCCAAGAGAGTGATCCTGGCCGACG
CTAATCTGGACAAAGTGCTGTCCGCCTACAACAAGCACCGGGATAAGCCCATCAGAGAGCAGGCCGAGAATA
TCATCCACCTGTTTACCCTGACCAATCTGGGAGCCCCTGCCGCCTTCAAGTACTTTGACACCACCATCGACCGGA
AGAGGTACACCAGCACCAAAGAGGTGCTGGACGCCACCCTGATCCACCAGAGCATCACCGGCCTGTACGAGA
CACGGATCGACCTGTCTCAGCTGGGAGGCGACAAAAGGCCGGCGGCCACGAAAAAGGCCGGCCAGGCAAAA
AAGAAAAAGTAA

## pPIK16045_pX330_Flag-SuperFi-Cas9 (Addgene #184370)

DNA sequence of the HiFiSpCas9 coding cassette:

Human codon optimized *S. pyogenes* Cas9 coloured in purple, NLS underlined, 3xFLAG tag in *light blue*, silent mutations underlined and marked with yellow.

ATG*GACTATAAGGACCACGACGGAGACTACAAGGATCATGATATTGATTACAAAGACGATGACGATAAG*ATG
GCCCCAAAGAAGAAGCGGAAGGTCGGTATCCACGGAGTCCCAGCAGCCGACAAGAAGTACAGCATCGGCCTG
GACATCGGCACCAACTCTGTGGGCTGGGCCGTGATCACCGACGAGTACAAGGTGCCCAGCAAGAAATTCAAG
GTGCTGGGCAACACCGACCGGCACAGCATCAAGAAGAACCTGATCGGAGCCCTGCTGTTCGACAGCGGCGAA
ACAGCCGAGGCCACCCGGCTGAAGAGAACCGCCAGAAGAAGATACACCAGACGGAAGAACCGGATCTGCTAT
CTGCAAGAGATCTTCAGCAACGAGATGGCCAAGGTGGACGACAGCTTCTTCCACAGACTGGAAGAGTCCTTCC
TGGTGGAAGAGGATAAGAAGCACGAGCGGCACCCCATCTTCGGCAACATCGTGGACGAGGTGGCCTACCACG
AGAAGTACCCCACCATCTACCACCTGAGAAAGAAACTGGTGGACAGCACCGACAAGGCCGACCTGCGGCTGA
TCTATCTGGCCCTGGCCCACATGATCAAGTTCCGGGGCCACTTCCTGATCGAGGGCGACCTGAACCCCGACAA
CAGCGACGTGGACAAGCTGTTCATCCAGCTGGTGCAGACCTACAACCAGCTGTTCGAGGAAAACCCCATCAAC
GCCAGCGGCGTGGACGCCAAGGCCATCCTGTCTGCCAGACTGAGCAAGAGCAGACGGCTGGAAAATCTGATC
GCCCAGCTGCCCGGCGAGAAGAAGAATGGCCTGTTCGGAAACCTGATTGCCCTGAGCCTGGGCCTGACCCCC
AACTTCAAGAGCAACTTCGACCTGGCCGAGGATGCCAAACTGCAGCTGAGCAAGGACACCTACGACGACGAC
CTGGACAACCTGCTGGCCCAGATCGGCGACCAGTACGCCGACCTGTTTCTGGCCGCCAAGAACCTGTCCGACG
CCATCCTGCTGAGCGACATCCTGAGAGTGAACACCGAGATCACCAAGGCCCCCCTGAGCGCCTCTATGATCAA
GAGATACGACGAGCACCACCAGGACCTGACCCTGCTGAAAGCTCTCGTGCGGCAGCAGCTGCCTGAGAAGTA
CAAAGAGATTTTCTTCGACCAGAGCAAGAACGGCTACGCCGGCTACATTGACGGCGGAGCCAGCCAGGAAGA
GTTCTACAAGTTCATCAAGCCCATCCTGGAAAAGATGGACGGCACCGAGGAACTGCTCGTGAAGCTGAACAGA
GAGGACCTGCTGCGGAAGCAGCGGACCTTCGACAACGGCAGCATCCCCCACCAGATCCACCTGGGAGAGCTG
CACGCCATTCTGCGGCGGCAGGAAGATTTTTACCCATTCCTGAAGGACAACCGGGAAAAGATCGAGAAGATCC
TGACCTTCCGCATCCCCTACTACGTGGGCCCTCTGGCCAGGGGAAACAGCAGATTCGCCTGGATGACCAGAAA
GAGCGAGGAAACCATCACCCCCTGGAACTTCGAGGAAGTGGTGGACAAGGGCGCTTCCGCCCAGAGCTTCAT
CGAGCGGATGACCAACTTCGATAAGAACCTGCCCAACGAGAAGGTGCTGCCCAAGCACAGCCTGCTGTACGA
GTACTTCACCGTGTATAACGAGCTGACCAAAGTGAAATACGTGACCGAGGGAATGAGAAAGCCCGCCTTCCTG
AGCGGCGAGCAGAAAAAGGCCATCGTGGACCTGCTGTTCAAGACCAACCGGAAAGTGACCGTGAAGCAGCTG
AAAGAGGACTACTTCAAGAAAATCGAGTGCTTCGACTCCGTGGAAATCTCCGGCGTGGAAGATCGGTTCAACG
CCTCCCTGGGCACATACCACGATCTGCTGAAAATTATCAAGGACAAGGACTTCCTGGACAATGAGGAAAACGA
GGACATTCTGGAAGATATCGTGCTGACCCTGACACTGTTTGAGGACAGAGAGATGATCGAGGAACGGCTGAA

AACCTATGCCCACCTGTTCGACGACAAAGTGATGAAGCAGCTGAAGCGGCGGAGATACACCGGCTGGGGCAG
GCTGAGCCGGAAGCTGATCAACGGCATCCGGGACAAGCAGTCCGGCAAGACAATCCTGGATTTCCTGAAGTC
CGACGGCTTCGCCAACAGAAACTTCATGCAGCTGATCCACGACGACAGCCTGACCTTTAAAGAGGACATCCAG
AAAGCCCAGGTGTCCGGCCAGGGCGATAGCCTGCACGAGCACATTGCCAATCTGGCCGGCAGCCCCGCCATT
AAGAAGGGCATCCTGCAGACAGTGAAGGTGGTGGACGAGCTCGTGAAAGTGATGGGCCGGCACAAGCCCGA
GAACATCGTGATCGAAATGGCCAGAGAGAACCAGACCACCCAGAAGGGACAGAAGAACAGCCGCGAGAGAA
TGAAGCGGATCGAAGAGGGCATCAAAGAGCTGGGCAGCCAGATCCTGAAAGAACACCCCGTGGAAAACACC
CAGCTGCAGAACGAGAAGCTGTACCTGTACTACCTGCAGAATGGGCGGGATATGTACGTGGACCAGGAACTG
GACATCAACCGGCTGTCCGACTACGATGTGGACCATATCGTGCCTCAGAGCTTTCTGAAGGACGACTCCATCG
ACAACAAGGTGCTGACCAGAAGCGACAAGAACCGGGGCAAGAGCGACAACGTGCCCTCCGAAGAGGTCGTG
AAGAAGATGAAGAACTACTGGCGGCAGCTGCTGAACGCCAAGCTGATTACCCAGAGAAAGTTCGACAATCTG
ACCAAGGCCGAGAGAGGCGGCCTGAGCGAACTGGATAAGGCCGGCTTCATCAAGAGACAGCTGGTGGAAAC
CCGGCAGATCACAAAGCACGTGGCACAGATCCTGGACTCCCGGATGAACACTAAGTACGACGAGAATGACAA
GCTGATCCGGGAAGTGAAAGTGATCACCCTGAAGTCCAAGCTGGTGTCCGATTTCCGGAAGGATTTCCAGTTT
TACAAAGTGCGCGAGATCAACAACTACCACCACGCCCACGACGCGTACCTGAACGCCGTCGTGGGAACCGCCC
TGATCAAAAAGTACCCTAAGCTGGAAAGCGAGTTCGTGGACGGCGACGACAAGGTGGACGACGACGACAAG
ATGATCGCCAAGAGCGAAGACGAAATCGGCGACGCTACCGCCAAGTACTTCTTCTACAGCAACATCATGAACT
TTTTCAAGACCGAGATTACCCTGGCCAACGGCGAGATCCGGAAGCGGCCTCTGATCGAGACAAACGGCGAAA
CCGGGGAGATCGTGTGGGATAAGGGCCGGGATTTTGCCACCGTGCGGAAAGTGCTGAGCATGCCCCAAGTGA
ATATCGTGAAAAAGACCGAGGTGCAGACAGGCGGCTTCAGCAAAGAGTCTATCCTGCCCAAGAGGAACAGCG
ATAAGCTGATCGCCAGAAAGAAGGACTGGGACCCTAAGAAGTACGGCGGCTTCGACAGCCCCACCGTGGCCT
ATTCTGTGCTGGTGGTGGCCAAAGTGGAAAAGGGCAAGTCCAAGAAACTGAAGAGTGTGAAAGAGCTGCTG
GGGATCACCATCATGGAAAGAAGCAGCTTCGAGAAGAATCCCATCGACTTTCTGGAAGCCAAGGGCTACAAA
GAAGTGAAAAAGGACCTGATCATCAAGCTGCCTAAGTACTCCCTGTTCGAGCTGGAAAACGGCCGGAAGAGA
ATGCTGGCCTCTGCCGGCGAACTGCAGAAGGGAAACGAACTGGCCCTGCCCTCCAAATATGTGAACTTCCTGT
ACCTGGCCAGCCACTATGAGAAGCTGAAGGGCTCCCCCGAGGATAATGAGCAGAAACAGCTGTTTGTGGAAC
AGCACAAGCACTACCTGGACGAGATCATCGAGCAGATCAGCGAGTTCTCCAAGAGAGTGATCCTGGCCGACG
CTAATCTGGACAAAGTGCTGTCCGCCTACAACAAGCACCGGGATAAGCCCATCAGAGAGCAGGCCGAGAATA
TCATCCACCTGTTTACCCTGACCAATCTGGGAGCCCCTGCCGCCTTCAAGTACTTTGACACCACCATCGACCGGA
AGAGGTACACCAGCACCAAAGAGGTGCTGGACGCCACCCTGATCCACCAGAGCATCACCGGCCTGTACGAGA
CACGGATCGACCTGTCTCAGCTGGGAGGCGACAAAAGGCCGGCGGCCACGAAAAAGGCCGGCCAGGCAAAA
AAGAAAAAGTAA

## px330-Flag-B-HypaR-SpCas9 (Addgene #126764)

DNA sequence of the HypaR-SpCas9 coding cassette:

Human codon optimized *S. pyogenes* Cas9 shown in purple, NLS underlined, modified codons in red, 3xFLAG tag in *light blue*, silent mutations underlined and marked with yellow, insertions marked with green, deletions crossed out and marked with grey.

ATG*GACTATAAGGACCACGACGGAGACTACAAGGATCATGATATTGATTACAAAGACGATGACGATAAG*ATG
GCCCCAAAGAAGAAGCGGAAGGTCGGTATCCACGGAGTCCCAGCAGCCGACAAGAAGTACAGCATCGGCCTG
GACATCGGCACCAACTCTGTGGGCTGGGCCGTGATCACCGACGAGTACAAGGTGCCCAGCAAGAAATTCAAG
GTGCTGGGCAACACCGACCGGCACAGCATCAAGAAGAACCTGATCGGAGCCCTGCTGTTCGACAGCGGCGAA
ACAGCCGAGGCCACCCGGCTGAAGAGAACCGCCAGAAGAAGATACACCAGACGGAAGAACCGGATCTGCTAT
CTGCAAGAGATCTTCAGCAACGAGATGGCCAAGGTGGACGACAGCTTCTTCCACAGACTGGAAGAGTCCTTCC
TGGTGGAAGAGGATAAGAAGCACGAGCGGCACCCCATCTTCGGCAACATCGTGGACGAGGTGGCCTACCACG
AGAAGTACCCCACCATCTACCACCTGAGAAAGAAACTGGTGGACAGCACCGACAAGGCCGACCTGCGGCTGA
TCTATCTGGCCCTGGCCCACATGATCAAGTTCCGGGGCCACTTCCTGATCGAGGGCGACCTGAACCCCGACAA
CAGCGACGTGGACAAGCTGTTCATCCAGCTGGTGCAGACCTACAACCAGCTGTTCGAGGAAAACCCCATCAAC
GCCAGCGGCGTGGACGCCAAGGCCATCCTGTCTGCCAGACTGAGCAAGAGCAGACGGCTGGAAAATCTGATC
GCCCAGCTGCCCGGCGAGAAGAAGAATGGCCTGTTCGGAAACCTGATTGCCCTGAGCCTGGGCCTGACCCCC
AACTTCAAGAGCAACTTCGACCTGGCCGAGGATGCCAAACTGCAGCTGAGCAAGGACACCTACGACGAC
CTGGACAACCTGCTGGCCCAGATCGGCGACCAGTACGCCGACCTGTTTCTGGCCGCCAAGAACCTGTCCGACG
CCATCCTGCTGAGCGACATCCTGAGAGTGAACACCGAGATCACCAAGGCCCCCCTGAGCGCCTCTATGATCAA
GAGATACGACGAGCACCACCAGGACCTGACCCTGCTGAAAGCTCTCGTGCGGCAGCAGCTGCCTGAGAAGTA
CAAAGAGATTTTCTTCGACCAGAGCAAGAACGGCTACGCCGGCTACATTGACGGCGGAGCCAGCCAGGAAGA
GTTCTACAAGTTCATCAAGCCCATCCTGGAAAAGATGGACGGCACCGAGGAACTGCTCGTGAAGCTGAACAGA
GAGGACCTGCTGCGGAAGCAGCGGACCTTCGACAACGGCAGCATCCCCCACCAGATCCACCTGGGAGAGCTG
CACGCCATTCTGCGGCGGCAGGAAGATTTTTACCCATTCCTGAAGGACAACCGGGAAAAGATCGAGAAGATCC
TGACCTTCCGCATCCCCTACTACGTGGGCCCTCTGGCCAGGGGAAACAGCAGATTCGCCTGGATGACCAGAAA
GAGCGAGGAAACCATCACCCCCTGGAACTTCGAGGAAGTGGTGGACAAGGGCGCTTCCGCCCAGAGCTTCAT
CGAGCGGATGACCAACTTCGATAAGAACCTGCCCAACGAGAAGGTGCTGCCCAAGCACAGCCTGCTGTACGA
GTACTTCACCGTGTATAACGAGCTGACCAAAGTGAAATACGTGACCGAGGGAATGAGAAAGCCCGCCTTCCTG
AGCGGCGAGCAGAAAAAGGCCATCGTGGACCTGCTGTTCAAGACCAACCGGAAAGTGACCGTGAAGCAGCTG
AAAGAGGACTACTTCAAGAAAATCGAGTGCTTCGACTCCGTGGAAATCTCCGGCGTGGAAGATCGGTTCAACG
CCTCCCTGGGCACATACCACGATCTGCTGAAAATTATCAAGGACAAGGACTTCCTGGACAATGAGGAAAACGA

GGACATTCTGGAAGATATCGTGCTGACCCTGACACTGTTTGAGGACAGAGAGATGATCGAGGAACGGCTGAA
AACCTATGCCCACCTGTTCGACGACAAAGTGATGAAGCAGCTGAAGCGGCGGAGATACACCGGCTGGGGCGC
GCTGAGCCGGAAGCTGATCAACGGCATCCGCGACAAGCAGTCCGGCAAGACAATCCTGGATTTCCTGAAGTCC
GACGGCTTCGCCAACAGAGCCTTTGCAGCCCTGATCGCTGACGACAGCCTGACCTTTAAAGAGGACATCCAGA
AAGCCCAGGTGTCCGGCCAGGGCGATAGCCTGCACGAGCACATTGCCAATCTGGCCGGCAGCCCCGCCATTA
AGAAGGGCATCCTGCAGACAGTGAAGGTGGTGGACGAGCTCGTGAAAGTGATGGGCCGGCACAAGCCCGAG
AACATCGTGATCGAAATGGCCAGAGAGAACCAGACCACCCAGAAGGGACAGAAGAACAGCCGCGAGAGAAT
GAAGCGGATCGAAGAGGGCATCAAAGAGCTGGGCAGCCAGATCCTGAAAGAACACCCCGTGGAAAACACCC
AGCTGCAGAACGAGAAGCTGTACCTGTACTACCTGCAGAATGGGCGGGATATGTACGTGGACCAGGAACTGG
ACATCAACCGGCTGTCCGACTACGATGTGGACCATATCGTGCCTCAGAGCTTTCTGAAGGACGACTCCATCGAC
AACAAGGTGCTGACCAGAAGCGACAAGAACCGGGGCAAGAGCGACAACGTGCCCTCCGAAGAGGTCGTGAA
GAAGATGAAGAACTACTGGCGGCAGCTGCTGAACGCCAAGCTGATTACCCAGAGAAAGTTCGACAATCTGAC
CAAGGCCGAGAGAGGCGGCCTGAGCGAACTGGATAAGGCCGGCTTCATCAAGAGACAGCTGGTGGAAACCC
GGCAGATCACAAAGCACGTGGCACAGATCCTGGACTCCCGGATGAACACTAAGTACGACGAGAATGACAAGC
TGATCCGGGAAGTGAAAGTGATCACCCTGAAGTCCAAGCTGGTGTCCGATTTCCGGAAGGATTTCCAGTTTTA
CAAAGTGCGCGAGATCAACAACTACCACCACGCCCACGACGCGTACCTGAACGCCGTCGTGGGAACCGCCCTG
ATCAAAAAGTACCCTAAGCTGGGTGGC~~GAAAGCGAGTTCGTGTACGGCGACTAC~~AAGGTGTACGACGTACGG
AAGATGATCGCCAAGAGCGAGCAGGAAATCGGCAAGGCTACCGCCAAGTACTTCTTCTACAGCAACATCATGA
ACTTTTTCAAGACCGAGATTACCCTGGCCAACGGCGAGATCCGGAAGCGGCCTCTGATCGAGACAAACGGCGA
AACCGGGGAGATCGTGTGGGATAAGGGCCGGGATTTTGCCACCGTGCGGAAAGTGCTGAGCATGCCCCAAGT
GAATATCGTGAAAAAGACCGAGGTGCAGACAGGCGGCTTCAGCAAAGAGTCTATCCTGCCCAAGAGGAACAG
CGATAAGCTGATCGCCAGAAAGAAGGACTGGGACCCTAAGAAGTACGGCGGCTTCGACAGCCCCACCGTGGC
CTATTCTGTGCTGGTGGTGGCCAAAGTGGAAAAGGGCAAGTCCAAGAAACTGAAGAGTGTGAAAGAGCTGCT
GGGGATCACCATCATGGAAAGAAGCAGCTTCGAGAAGAATCCCATCGACTTTCTGGAAGCCAAGGGCTACAA
AGAAGTGAAAAAGGACCTGATCATCAAGCTGCCTAAGTACTCCCTGTTCGAGCTGGAAAACGGCCGGAAGAG
AATGCTGGCCTCTGCCGGCGAACTGCAGAAGGGAAACGAACTGGCCCTGCCCTCCAAATATGTGAACTTCCTG
TACCTGGCCAGCCACTATGAGAAGCTGAAGGGCTCCCCCGAGGATAATGAGCAGAAACAGCTGTTTGTGGAA
CAGCACAAGCACTACCTGGACGAGATCATCGAGCAGATCAGCGAGTTCTCCAAGAGAGTGATCCTGGCCGAC
GCTAATCTGGACAAAGTGCTGTCCGCCTACAACAAGCACCGGGATAAGCCCATCAGAGAGCAGGCCGAGAAT
ATCATCCACCTGTTTACCCTGACCAATCTGGGAGCCCCTGCCGCCTTCAAGTACTTTGACACCACCATCGACCGG
AAGAGGTACACCAGCACCAAAGAGGTGCTGGACGCCACCCTGATCCACCAGAGCATCACCGGCCTGTACGAG
ACACGGATCGACCTGTCTCAGCTGGGAGGCGACAAAAGGCCGGCGGCCACGAAAAAGGCCGGCCAGGCAAA
AAAGAAAAAGTAA

## SuperFi mutants plasmids

SuperFi-Cas9 mutant coding plasmids were constructed from pPIK16045_pX330_Flag-SuperFi-Cas9 (#184370) plasmid. The plasmid was digested with Eco72I and Mva1269I restriction enzymes and assembled with two PCR fragments using the NEBuilder HiFi DNA Assembly Master Mix. The PCR fragments were generated from the same plasmid using the following primers:

Frament 1: fwd primer: GGAAACCCGGCAGATCACAAAGCACGTGGCACA
Frament 2: rev primer: cagttcgccggcagaggc

SuperFi (D1010Y):
Fragment 1 rev: CACGAACTCGCTTTCCAGcttaggg
Fragment 2 fwd: aGCTGGAAAGCGAGTTCGTGtacGGCGACgacAAGGTGg

SuperFi (D1013Y):
Fragment 1 rev: CACGAACTCGCTTTCCAGcttaggg
Fragment 2 fwd: aGCTGGAAAGCGAGTTCGTGgacGGCGACtACAAGGTGGACGACGACGACaaGa

SuperFi (D1016Y):
Fragment 1 rev: CACCTTgtcGTCGCCgtcCACG
Fragment 2 fwd: GgacGGCGACgacAAGGTGTacGACgacgacAAGATGATCGcc

SuperFi (D1018V):
Fragment 1 rev: CACCTTgtcGTCGCCgtcCACG
Fragment 2 fwd: GgacGGCGACgacAAGGTGgacGACgtagacAAGATGATCGCCAAGAGCGaag

SuperFi (D1019R):
Fragment 1 rev: CACCTTgtcGTCGCCgtcCACG
Fragment 2 fwd: GgacGGCGACgacAAGGTGgacGACgaccggAAGATGATCGCCAAGAGCGAagacG

SuperFi (D1027Q):
Fragment 1 rev: TCGCTCTTGGCGATCATCTTgtcg
Fragment 2 fwd: AAGATGATCGCCAAGAGCGAgcagGAAATCGGCgacGCTACCGC

SuperFi (D1031K):

Fragment 1 rev: TCGCTCTTGGCGATCATCTTgtcg

Fragment 2 fwd:

AAGATGATCGCCAAGAGCGAagacGAAATCGGCaagGCTACCGCCAAGTACTTCTTCTACAG

## Supplementary Note 3: SpCas9 variants, bacterial expression plasmids

Bacterial expression plasmids expressing SpCas9-HF1 and SuperFi-Cas9 were constructed from pET-Cas9-NLS-6xHis (Addgene #62933)[3] plasmid in two steps. In the first step, the plasmid was digested with NcoI and PacI restriction enzymes and assembled with one PCR fragment using the NEBuilder HiFi DNA Assembly Master Mix. The PCR fragment was generated from pX330-Flag-WT_SpCas9 (without sgRNA; with silent mutations) (Addgene #126753) using the following primers: TCTAGAAATAATTTTGTTTAACTTTAAGAAGGAGATATACCATGGACAAGAAGTACAGCATCGGC and GGTGGCAGCAGCCTAGGTTATTAGTGATGGTGGTGATGCTTATCCATCTTTTTCTTTTTGCCTGGCCGG.
SpCas9-HF1: In the second step, the first step product was digested with BglII and Eco52I restriction enzymes and assembled with one PCR fragment using the NEBuilder HiFi DNA Assembly Master Mix. The PCR fragment was generated from pX330-Flag-SpCas9-HF1 (without sgRNA; with silent mutations) (#126755) using the following primers:
GATACACCAGACGGAAGAACCGGATCTGCTATCTGCAAGA and CTTCTGCAGTTCGCCGGCAGAGGCC.
SuperFi-Cas9: In the second step, the first step product was digested with Eco72I and Mva1269I restriction enzymes and assembled with one PCR fragment using the NEBuilder HiFi DNA Assembly Master Mix. The PCR fragment was generated from pPIK16045_pX330_Flag-SuperFi-Cas9 (#184370) using the following primers:
GGAAACCCGGCAGATCACAAAGCACGTGGCACA and cagttcgccggcagaggc.

## Supplementary Note 4: Other plasmid sequences

### *Prnp*.HA-EGFP-DHFR[DD]

1,000 bp-long homology arm to the *Prnp* gene-EGFP-DHFR[DD] [EGFP-folA dihydrofolate reductase destabilization domain] fusion protein coding cassette-1,000 bp-long homology arm to the *Prnp* gene. Cloned from pBMN DHFR(DD)-YFP (#29325)[4] using standard molecular biology techniques

Plasmid sequence:

ACATGTGAAGACAACTACTTGTGAATGCAGCTAGATGTATCTCAGCAGCCCAGCCCGCCCGGCTCTCTTGTCTA
TGGAGAGAGTGAGGAATTCTCAAGTGAGCCTGCCAAAACTCTAGATGTTTCCTGTCTCTGATAAACTTAGGTTG

AAAATTCCCTCAAGGAGATTCTTGGCTTTGTGCTTAGGGGATGTTAATTCCGTCACTTGACAGCTGTGTTGTGT
CCTCCTCTGTGCCAGGCACTGCCCTTACCCATAAAATATGGCAACGAAACAGAGGCTCTTGGTTTGGTTTGGAT
TCTGGGGCATGAGCTGTAAAGCCCAGATGTATTAGAACTCACAGCCGTCCTGTTTCAGCCTCTACTTCCCAAGT
GCTGGGGCACCAACGTGCACTTCCTCATGCCTGGCTCTGGAGACCTACTGCTTGTCTCCAGGGCTCAAACACTG
AGTCAGCTTTCTTCAAGTCCTTGCTCCTGCTGTAGCCACTCAGGAGCCCTCCTGACTAGACCATGACTCAGGCCC
TTGTGGTGTTACGGTTACTCAGGACCAGTGTACTCACAGCTACCCCTGCAGGTGACTTTCTGCATTCTGGGGAA
TGAAGCCTACATCCGTGGATAAAGGTTCTCCTCTGTGTAGAGGCTCACACCCCACAGGACCCTGGGGCCATTAT
AGCAGCCTTATAGTACAGCTGCCAGGCTCCCCACAAGATCATGCCCATTTCCAAATTCCACTACATTGTTAAAG
CTCAAAGCCATGGCGTAACAACCATGCAATATCACCTAGACCAGACGTGGTTTACCAGTTGGGGTAACTCTTGT
CAAATCTGTCCTCAGAGGATGGGATGAGCTGTGTGTTTTTGATTTACTTTTTTCCTGAAGGAAAAGCTACGGGG
GGGGGGGGGCGGGGGGGAGGGTTGACGCCATGACTTTCATACATTTGCTTTGTAGATAGATGTCAAGGACC
TTCAGCCTAAATACTGGGCACTGATACCTTGTTCCTCATTTTGCAGATCAGTCATCACACCTGTCTTCATTAATAC
CGGTCGCCACCATGGTGAGCAAGGGCGAGGAGCTGTTCACCGGGGTGGTGCCCATCCTGGTCGAGCTGGACG
GCGACGTAAACGGCCACAAGTTCAGCGTGTCCGGCGAGGGCGAGGGCGATGCCACCTACGGCAAGCTGACCC
TGAAGTTCATCTGCACCACCGGCAAGCTGCCCGTGCCCTGGCCCACCCTCGTGACCACCCTGACCTACGGCGTG
CAGTGCTTCAGCCGCTACCCCGACCACATGAAGCAGCACGACTTCTTCAAGTCCGCCATGCCCGAAGGCTACGT
CCAGGAGCGCACCATCTTCTTCAAGGACGACGGCAACTACAAGACCCGCGCCGAGGTGAAGTTCGAGGGCGA
CACCCTGGTGAACCGCATCGAGCTGAAGGGCATCGACTTCAAGGAGGACGGCAACATCCTGGGGCACAAGCT
GGAGTACAACTACAACAGCCACAACGTCTATATCATGGCCGACAAGCAGAAGAACGGCATCAAGGTGAACTTC
AAGATCCGCCACAACATCGAGGACGGCAGCGTGCAGCTCGCCGACCACTACCAGCAGAACACCCCCATCGGC
GACGGCCCCGTGCTGCTGCCCGACAACCACTACCTGAGCACCCAGTCCGCCCTGAGCAAAGACCCCAACGAGA
AGCGCGATCACATGGTCCTGCTGGAGTTCGTGACCGCCGCCGGGATCACTCTCGGCATGGACGAGCTGTACAA
GGGATCCCTTGCAGAGGCGTCTGCTAAAGCGGCAGCGGGCTCGAGTATCAGTCTGATTGCGGCGTTAGCGGT
AGATTACGTTATCGGCATGGAAAACGCCATGCCGTGGAACCTGCCTGCCGATCTCGCCTGGTTTAAACGCAAC
ACCTTAAATAAACCCGTGATTATGGGCCGCCATACCTGGGAATCAATCGGTCGTCCGTTGCCAGGACGCAAAA
ATATTATCCTCAGCAGTCAACCGAGTACGGACGATCGCGTAACGTGGGTGAAGTCGGTGGATGAAGCCATCG
CGGCGTGTGGTGACGTACCAGAAATCATGGTGATTGGCGGCGGTCGCGTTATTGAACAGTTCTTGCCAAAAGC
GCAAAAACTGTATCTGACGCATATCGACGCAGAAGTGGAAGGCGACACCCATTTCCCGGATTACGAGCCGGAT
GACTGGGAATCGGTATTCAGCGAATTCCACGATGCTGATGCGCAGAACTCTCACAGCTATTGCTTTGAGATTCT
GGAGCGGCGATAGCAATTGTTGTTGTTAACTTGTTTATTGCAGCTTATAATGGTTACAAATAAAGCAATAGCAT
CACAAATTTCACAAATAAAGCATTTTTTTCACTGCATTCTAGTTGTGGTTTGTCCAAACTCATCAATGTATCTTAA
CGCGTCGTCTCTCCTCGGGAGGCCTTCCTGCTTGTTCCTTCGCATTCTCGTGGTCTAGGCTGGGGGAGGGGTTA
TCCACCTGTAGCTCTTTCAATTGAGGTGGTTCTCATTCTTGCTTCTCTGTGTCCCCCATAGGCTAATACCCCTGGC
ACTGATGGGCCCTGGGAAATGTACAGTAGACCAGTTGCTCTTTGCTTCAGGTCCCTTTGATGGAGTCTGTCATC
AGCCAGTGCTAACACCGGGCCAATAAGAATATAACACCAAATAACTGCTGGCTAGTTGGGGCTTTGTTTTGGT

CTAGTGAATAAATACTGGTGTATCCCCTGACTTGTACCCAGAGTACAAGGTGACAGTGACACATGTAACTTAGC
ATAGGCAAAGGGTTCTACAACCAAAGAAGCCACTGTTTGGGGATGGCGCCCTGGAAAACAGCCTCCCACCTG
GGATAGCTAGAGCATCCACACGTGGAATTCTTTCTTTACTAACAAACGATAGCTGATTGAAGGCAACAGGAAA
AAAAAAATCAAATTGTCCTACTGACGTTGAAAGCAAACCTTTGTTCATTCCCAGGGCACTAGAATGATCTTTAG
CCTTGCTTGGATTGAACTAGGAGATCTTGACTCTGAGGAGAGCCAGCCCTGTAAAAAGCTTGGTCCTCCTGTG
ACGGGAGGGATGGTTAAGGTACAAAGGCTAGAAACTTGAGTTTCTTCATTTCTGTCTCACAATTATCAAAAGCT
AGAATTAGCTTCTGCCCTATGTTTCTGTACTTCTATTTGAACTGGATAACAGAGAGACAATCTAAACATTCTCTT
AGGCTGCAGATAAGAGAAGTAGGCTCCATTCCAAAGTGGGAAAGAAATTCTGCTAGCATTGTTTAAATCAGGC
AAAATTTGTTCCTGAAGTTGCTTTTTACCCCAGCAGACATAAACTGCGATAGCTTCAGCTTGCACTGTGGATTTT
CTGTATAGAATATATAAAACATAACTTCAAGCTTATGTCTTCTTTTTAAAACATCTGAAGTATGGGACGCCCTTT
CTCGAGACGCACAATGTGAACCATCACCCTAATCAAGTTTTTTGGGGTCGAGGTGCCGTAAAGCACTAAATCG
GAACCCTAAAGGGAGCCCCCGATTTAGAGCTTGACGGGGAAAGCCGGCGAACGTGGCGAGAAAGGAAGGGA
AGAAAGCGAAAGGAGCGGGCGCTAGGGCGCTGGCAAGTGTAGCGGTCACGCTGCGCGTAACCACCACACCC
GCCGCGCTTAATGCGCCGCTACAGGGCGCGTCAGGTGGCACTTTTCGGGGAAATGTGCGCGGAACCCCTATTT
GTTTATTTTTCTAAATACATTCAAATATGTATCCGCTCATGAGACAATAACCCTGATAAATGCTTCAATAATATT
GAAAAAGGAAGAGTCCTGAGGCGGAAAGAACCAGCTGTGGAATGTGTGTCAGTTAGGGTGTGGAAAGTCCC
CAGGCTCCCCAGCAGGCAGAAGTATGCAAAGCATGCATCTCAATTAGTCAGCAACCAGGTGTGGAAAGTCCCC
AGGCTCCCCAGCAGGCAGAAGTATGCAAAGCATGCATCTCAATTAGTCAGCAACCATAGTCCCGCCCCTAACTC
CGCCCATCCCGCCCCTAACTCCGCCCAGTTCCGCCCATTCTCCGCCCCATGGCTGACTAATTTTTTTTATTTATGC
AGAGGCCGAGGCCGCCTCGGCCTCTGAGCTATTCCAGAAGTAGTGAGGAGGCTTTTTTGGAGGCCTAGGCTTT
TGCAAAGATCGATCAAGAGACAGGATGAGGATCGTTTCGCATGATTGAACAAGATGGATTGCACGCAGGTTC
TCCGGCCGCTTGGGTGGAGAGGCTATTCGGCTATGACTGGGCACAACAGACAATCGGCTGCTCTGATGCCGCC
GTGTTCCGGCTGTCAGCGCAGGGGCGCCCGGTTCTTTTTGTCAAGACCGACCTGTCCGGTGCCCTGAATGAAC
TGCAAGACGAGGCAGCGCGGCTATCGTGGCTGGCCACGACGGGCGTTCCTTGCGCAGCTGTGCTCGACGTTG
TCACTGAAGCGGGAAGGGACTGGCTGCTATTGGGCGAAGTGCCGGGGCAGGATCTCCTGTCATCTCACCTTGC
TCCTGCCGAGAAAGTATCCATCATGGCTGATGCAATGCGGCGGCTGCATACGCTTGATCCGGCTACCTGCCCAT
TCGACCACCAAGCGAAACATCGCATCGAGCGAGCACGTACTCGGATGGAAGCCGGTCTTGTCGATCAGGATG
ATCTGGACGAAGAGCATCAGGGGCTCGCGCCAGCCGAACTGTTCGCCAGGCTCAAGGCGAGCATGCCCGACG
GCGAGGATCTCGTCGTGACCCATGGCGATGCCTGCTTGCCGAATATCATGGTGGAAAATGGCCGCTTTTCTGG
ATTCATCGACTGTGGCCGGCTGGGTGTGGCGGACCGCTATCAGGACATAGCGTTGGCTACCCGTGATATTGCT
GAAGAGCTTGGCGGCGAATGGGCTGACCGCTTCCTCGTGCTTTACGGTATCGCCGCTCCCGATTCGCAGCGCA
TCGCCTTCTATCGCCTTCTTGACGAGTTCTTCTGAGCGGGACTCTGGGGTTCGAAATGACCGACCAAGCGACGC
CCAACCTGCCATCACGAGATTTCGATTCCACCGCCGCCTTCTATGAAAGGTTGGGCTTCGGAATCGTTTTCCGG
GACGCCGGCTGGATGATCCTCCAGCGCGGGGATCTCATGCTGGAGTTCTTCGCCCACCCTAGGGGGAGGCTA
ACTGAAACACGGAAGGAGACAATACCGGAAGGAACCCGCGCTATGACGGCAATAAAAAGACAGAATAAAAC

GCACGGTGTTGGGTCGTTTGTTCATAAACGCGGGGTTCGGTCCCAGGGCTGGCACTCTGTCGATACCCCACCG
AGACCCCATTGGGGCCAATACGCCCGCGTTTCTTCCTTTTCCCCACCCCACCCCCCAAGTTCGGGTGAAGGCCC
AGGGCTCGCAGCCAACGTCGGGGCGGCAGGCCCTGCCATAGCCTCAGGTTACTCATATATACTTTAGATTGAT
TTAAAACTTCATTTTTAATTTAAAAGGATCTAGGTGAAGATCCTTTTTGATAATCTCATGACCAAAATCCCTTAA
CGTGAGTTTTCGTTCCACTGAGCGTCAGACCCCGTAGAAAAGATCAAAGGATCTTCTTGAGATCCTTTTTTTCTG
CGCGTAATCTGCTGCTTGCAAACAAAAAAACCACCGCTACCAGCGGTGGTTTGTTTGCCGGATCAAGAGCTAC
CAACTCTTTTTCCGAAGGTAACTGGCTTCAGCAGAGCGCAGATACCAAATACTGTCCTTCTAGTGTAGCCGTAG
TTAGGCCACCACTTCAAGAACTCTGTAGCACCGCCTACATACCTCGCTCTGCTAATCCTGTTACCAGTGGCTGCT
GCCAGTGGCGATAAGTCGTGTCTTACCGGGTTGGACTCAAGACGATAGTTACCGGATAAGGCGCAGCGGTCG
GGCTGAACGGGGGGTTCGTGCACACAGCCCAGCTTGGAGCGAACGACCTACACCGAACTGAGATACCTACAG
CGTGAGCTATGAGAAAGCGCCACGCTTCCCGAAGGGAGAAAGGCGGACAGGTATCCGGTAAGCGGCAGGGT
CGGAACAGGAGAGCGCACGAGGGAGCTTCCAGGGGGAAACGCCTGGTATCTTTATAGTCCTGTCGGGTTTCG
CCACCTCTGACTTGAGCGTCGATTTTTGTGATGCTCGTCAGGGGGGCGGAGCCTATGGAAAAACGCCAGCAAC
GCGGCCTTTTTACGGTTCCTGGCCTTTTGCTGGCCTTTTGCTC

## *Sprn*.HA-CMV-EGFP

1,000 bp-long homology arm to the *Sprn* gene-CMV-EGFP protein coding cassette-1,000 bp-long homology arm to the Shadoo (*Sprn)* gene.

Plasmid sequence:
ACATGTGAAGACAACTAACAAAATCCAGTCGTGAGCTCTGCCTAAAGAAAAGGGTCCTCGCTGCCGCACCTTT
CCGCTTGGCCGGTCAAGGCCCCTAAATCGCTATCCGACCTAGGCTTGTGACCAGTAAGTAGGGGAGGTGGAT
GAACCCAGTGATGCTGGGAGGGAGGGGGAGGGGAGAGAGGACCTGAGGAGGATGGAGCTGCCGCCACCGA
GATGGCTGGTCCACAGCCAGCCGGAACCCATCCTGATCGGTTTACCTGTCCAGGATACTGCCCTGGACAAACC
CAAAAAGGGTGGAGCTGGAGCGGGGAAGAGACGTAATTACCTAGGGTCTGGGCCTGCTGGGCGGTTCACCC
ATCGCTAGTTGTTTGCGTTGCAAGGAATCGCTGATCTGGATTCTGACCCCCACCCTCACCCAGTGCATTCAGCC
GCAGCCACTGGCTGAAAGACTATCTCTTAAGGCATCAGGAGATCCAGATGCCAGAGCAAAAGTCACAAGGCT
GTCCATTCTAACCATAATCTGGGGGTATTGAAGGCTCTCCATTCCAAACTAGAATCCTAATCCACTAATCCATCC
CCTTCCAGAGACTCGTGTGCAGAGCGGGGGATTTGTGCCCCCCCCCAGGCCTGAGCCCCACTGTAGGAGCTC
CGCAAACCCCATTCTGGGACCCATCTCCACCCTATCACAATAGTAAAACGGCCGCCCTGTAGTTAAACCCTTTCC
CCACCACCCCATCTCCACCTGGTTATACAGCAGGAACCCAAAACCAAAGTCCTGGTGCTGGAGTTTAAAGAACC
CCTTCCCCAACCCTGAGCCCACTGTCCTCCAAGATGCTGGGAGCCCTCTATCGGATTACCACAAAACCAGAGGC
TGAAGTAGGTGTCCCCAGGTCCAGATGAGCCTATTCCCAAGCCCTGATACCCTCTTGCCCTGGTCCTAAACCAC
GCTCCACCCCTGCACAGAAGCTGAAGCCCCTTCCACCCTCTTCTCGCAGATTCTGCCCAGTAGGACACCTGTCTT

CATTAATAGTAATCAATTACGGGGTCATTAGTTCATAGCCCATATATGGAGTTCCGCGTTACATAACTTACGGT
AAATGGCCCGCCTGGCTGACCGCCCAACGACCCCCGCCCATTGACGTCAATAATGACGTATGTTCCCATAGTAA
CGCCAATAGGGACTTTCCATTGACGTCAATGGGTGGAGTATTTACGGTAAACTGCCCACTTGGCAGTACATCA
AGTGTATCATATGCCAAGTACGCCCCCTATTGACGTCAATGACGGTAAATGGCCCGCCTGGCATTATGCCCAGT
ACATGACCTTATGGGACTTTCCTACTTGGCAGTACATCTACGTATTAGTCATCGCTATTACCATGGTGATGCGGT
TTTGGCAGTACATCAATGGGCGTGGATAGCGGTTTGACTCACGGGGATTTCCAAGTCTCCACCCCATTGACGTC
AATGGGAGTTTGTTTTGGCACCAAAATCAACGGGACTTTCCAAAATGTCGTAACAACTCCGCCCCATTGACGCA
AATGGGCGGTAGGCGTGTACGGTGGGAGGTCTATATAAGCAGAGCTGGTTTAGTGAACCGTCAGATCCGCTA
GCGCTACCGGTCGCCACCATGGTGAGCAAGGGCGAGGAGCTGTTCACCGGGGTGGTGCCCATCCTGGTCGAG
CTGGACGGCGACGTAAACGGCCACAAGTTCAGCGTGTCCGGCGAGGGCGAGGGCGATGCCACCTACGGCAA
GCTGACCCTGAAGTTCATCTGCACCACCGGCAAGCTGCCCGTGCCCTGGCCCACCCTCGTGACCACCCTGACCT
ACGGCGTGCAGTGCTTCAGCCGCTACCCCGACCACATGAAGCAGCACGACTTCTTCAAGTCCGCCATGCCCGA
AGGCTACGTCCAGGAGCGCACCATCTTCTTCAAGGACGACGGCAACTACAAGACCCGCGCCGAGGTGAAGTTC
GAGGGCGACACCCTGGTGAACCGCATCGAGCTGAAGGGCATCGACTTCAAGGAGGACGGCAACATCCTGGG
GCACAAGCTGGAGTACAACTACAACAGCCACAACGTCTATATCATGGCCGACAAGCAGAAGAACGGCATCAA
GGTGAACTTCAAGATCCGCCACAACATCGAGGACGGCAGCGTGCAGCTCGCCGACCACTACCAGCAGAACAC
CCCCATCGGCGACGGCCCCGTGCTGCTGCCCGACAACCACTACCTGAGCACCCAGTCCGCCCTGAGCAAAGAC
CCCAACGAGAAGCGCGATCACATGGTCCTGCTGGAGTTCGTGACCGCCGCCGGGATCACTCTCGGCATGGAC
GAGCTGTACAAGTCCGGACTCAGATCTCGAGCTCAAGCTTCGAATTCTGCAGTCGACGGTACCGCGGGCCCGG
GATCCACCGGATCTAGATAACTGATCATAATCAGCCATACCACATTTGTAGAGGTTTTACTTGCTTTAAAAAACC
TCCCACACCTCCCCCTGAACCTGAAACATAAAATGAATGCAATTGTTGTTGTTAACTTGTTTATTGCAGCTTATA
ATGGTTACAAATAAAGCAATAGCATCACAAATTTCACAAATAAAGCATTTTTTTCACTGCATTCTAGTTGTGGTT
TGTCCAAACTCATCAATGTATCTTAACGCGTCGTCTCTCCTCACCAGGCTAAACTCCATCCCAGGTCTAGCTCCT
AGCCTGTCTTAAGGCCCCTAGGGCCCACCCTAATGGCCTCCTGCCCAGGGGGTAACTTCATTTTGCTAACTATG
ATTCCCCAGCCCAGAGAAGAGTCTGGCCATTCTGGGCCCACAAGGCCTCTTGCAATTTGTGGATGTAGTTTACT
CTCTTGCCCATCCCAGTTTCCCAGTCTTCCACTCTGGCAGGAACTAACAGCCCTACTACCAGAGAGGCAGGCTG
CCCTTGACTCTCCAGAACTGCCCAAGCAGGCATGCCTGTCTGCTTGCCCACCCTGACACGAGGACATGAACAAT
GCTGACCGCCAAGAAGAGGCCATCCTTGGGTGGGCCCGTCTGATTCTGCCACTAAAGCCCCCAAAGACCTTGA
GCCTCCCATGGAACCAGAGGGACTTATAGTTGATGAAGAGAGCATTCACTTAGACTGCAGTGTGAGAAATGTC
AGGTGCTAACCACCTGCCCAGAGCAGGTCCCAATGATGTCATAGCTCAGAAAACATCCAGAGCAGTAAAATAA
CCATCCCAGGACGCCCACCATGCTCCTCCTACAACATGCTACCAAAGCCAAGTAGTATGTTTCTTCCTGGCAGA
CTGCCTAAGAGACCTTGTGTCAGAAGGGTTTCCACTTGAAGCCACTTGGTCCTAAAATCCACTGAGGGTAGAG
GTTTTGAATACACTTTCAAAAACATTCCATTCTGCTTGAGCTTAAGGGTCATGAGTGAGGGTCACTTGGATATA
ATACCAATCCTGCTGGGGCCTTCTTTGTATATAACCCAAACTGCAAGATTCCCATAGTTCCAGTAGATAGCAGC
ATTTTATGTTGGGAGACCCCTCCCTTGGAAACGGTTGGACGGGGTTGGGGTGGGGAGGTGAGACAGAGCATG

GCACAGCTGACAGCTGGCAAACTGAACAGTGGAAGGGGCAGCAGATCTACAGCCCCACTGTGCCAGAACAAA

GCTAGCAGACAGATTCTCGAGACGCACTACGTGAACCATCACCCTAATCAAGTTTTTTGGGGTCGAGGTGCCG

TAAAGCACTAAATCGGAACCCTAAAGGGAGCCCCCGATTTAGAGCTTGACGGGGAAAGCCGGCGAACGTGGC

GAGAAAGGAAGGGAAGAAAGCGAAAGGAGCGGGCGCTAGGGCGCTGGCAAGTGTAGCGGTCACGCTGCGC

GTAACCACCACACCCGCCGCGCTTAATGCGCCGCTACAGGGCGCGTCAGGTGGCACTTTTCGGGGAAATGTGC

GCGGAACCCCTATTTGTTTATTTTTCTAAATACATTCAAATATGTATCCGCTCATGAGACAATAACCCTGATAAA

TGCTTCAATAATATTGAAAAAGGAAGAGTCCTGAGGCGGAAAGAACCAGCTGTGGAATGTGTGTCAGTTAGG

GTGTGGAAAGTCCCCAGGCTCCCCAGCAGGCAGAAGTATGCAAAGCATGCATCTCAATTAGTCAGCAACCAGG

TGTGGAAAGTCCCCAGGCTCCCCAGCAGGCAGAAGTATGCAAAGCATGCATCTCAATTAGTCAGCAACCATAG

TCCCGCCCCTAACTCCGCCCATCCCGCCCCTAACTCCGCCCAGTTCCGCCCATTCTCCGCCCCATGGCTGACTAA

TTTTTTTTATTTATGCAGAGGCCGAGGCCGCCTCGGCCTCTGAGCTATTCCAGAAGTAGTGAGGAGGCTTTTTT

GGAGGCCTAGGCTTTTGCAAAGATCGATCAAGAGACAGGATGAGGATCGTTTCGCATGATTGAACAAGATGG

ATTGCACGCAGGTTCTCCGGCCGCTTGGGTGGAGAGGCTATTCGGCTATGACTGGGCACAACAGACAATCGG

CTGCTCTGATGCCGCCGTGTTCCGGCTGTCAGCGCAGGGGCGCCCGGTTCTTTTTGTCAAGACCGACCTGTCCG

GTGCCCTGAATGAACTGCAAGACGAGGCAGCGCGGCTATCGTGGCTGGCCACGACGGGCGTTCCTTGCGCAG

CTGTGCTCGACGTTGTCACTGAAGCGGGAAGGGACTGGCTGCTATTGGGCGAAGTGCCGGGGCAGGATCTCC

TGTCATCTCACCTTGCTCCTGCCGAGAAAGTATCCATCATGGCTGATGCAATGCGGCGGCTGCATACGCTTGAT

CCGGCTACCTGCCCATTCGACCACCAAGCGAAACATCGCATCGAGCGAGCACGTACTCGGATGGAAGCCGGTC

TTGTCGATCAGGATGATCTGGACGAAGAGCATCAGGGGCTCGCGCCAGCCGAACTGTTCGCCAGGCTCAAGG

CGAGCATGCCCGACGGCGAGGATCTCGTCGTGACCCATGGCGATGCCTGCTTGCCGAATATCATGGTGGAAA

ATGGCCGCTTTTCTGGATTCATCGACTGTGGCCGGCTGGGTGTGGCGGACCGCTATCAGGACATAGCGTTGGC

TACCCGTGATATTGCTGAAGAGCTTGGCGGCGAATGGGCTGACCGCTTCCTCGTGCTTTACGGTATCGCCGCTC

CCGATTCGCAGCGCATCGCCTTCTATCGCCTTCTTGACGAGTTCTTCTGAGCGGGACTCTGGGGTTCGAAATGA

CCGACCAAGCGACGCCCAACCTGCCATCACGAGATTTCGATTCCACCGCCGCCTTCTATGAAAGGTTGGGCTTC

GGAATCGTTTTCCGGGACGCCGGCTGGATGATCCTCCAGCGCGGGGATCTCATGCTGGAGTTCTTCGCCCACC

CTAGGGGGAGGCTAACTGAAACACGGAAGGAGACAATACCGGAAGGAACCCGCGCTATGACGGCAATAAAA

AGACAGAATAAAACGCACGGTGTTGGGTCGTTTGTTCATAAACGCGGGGTTCGGTCCCAGGGCTGGCACTCTG

TCGATACCCCACCGAGACCCCATTGGGGCCAATACGCCCGCGTTTCTTCCTTTTCCCCACCCCACCCCCCAAGTT

CGGGTGAAGGCCCAGGGCTCGCAGCCAACGTCGGGGCGGCAGGCCCTGCCATAGCCTCAGGTTACTCATATA

TACTTTAGATTGATTTAAAACTTCATTTTTAATTTAAAAGGATCTAGGTGAAGATCCTTTTTGATAATCTCATGAC

CAAAATCCCTTAACGTGAGTTTTCGTTCCACTGAGCGTCAGACCCCGTAGAAAAGATCAAAGGATCTTCTTGAG

ATCCTTTTTTTCTGCGCGTAATCTGCTGCTTGCAAACAAAAAAACCACCGCTACCAGCGGTGGTTTGTTTGCCGG

ATCAAGAGCTACCAACTCTTTTTCCGAAGGTAACTGGCTTCAGCAGAGCGCAGATACCAAATACTGTTCTTCTA

GTGTAGCCGTAGTTAGGCCACCACTTCAAGAACTCTGTAGCACCGCCTACATACCTCGCTCTGCTAATCCTGTTA

CCAGTGGCTGCTGCCAGTGGCGATAAGTCGTGTCTTACCGGGTTGGACTCAAGACGATAGTTACCGGATAAGG

CGCAGCGGTCGGGCTGAACGGGGGGTTCGTGCACACAGCCCAGCTTGGAGCGAACGACCTACACCGAACTGA

GATACCTACAGCGTGAGCTATGAGAAAGCGCCACGCTTCCCGAAGGGAGAAAGGCGGACAGGTATCCGGTAA

GCGGCAGGGTCGGAACAGGAGAGCGCACGAGGGAGCTTCCAGGGGGAAACGCCTGGTATCTTTATAGTCCT

GTCGGGTTTCGCCACCTCTGACTTGAGCGTCGATTTTTGTGATGCTCGTCAGGGGGGCGGAGCCTATGGAAAA

ACGCCAGCAACGCGGCCTTTTTACGGTTCCTGGCCTTTTGCTGGCCTTTTGCTC


1 Kulcsar, P. I. *et al.* Blackjack mutations improve the on-target activities of increased fidelity variants of SpCas9 with 5'G-extended sgRNAs. *Nat Commun* **11**, 1223, doi:10.1038/s41467-020-15021-5 (2020).

2 Engler, C., Kandzia, R. & Marillonnet, S. A One Pot, One Step, Precision Cloning Method with High Throughput Capability. *Plos One* **3**, doi:ARTN e3647

10.1371/journal.pone.0003647 (2008).

3 Zuris, J. A. *et al.* Cationic lipid-mediated delivery of proteins enables efficient protein-based genome editing in vitro and in vivo. *Nat Biotechnol* **33**, 73-80, doi:10.1038/nbt.3081 (2015).

4 Iwamoto, M., Bjorklund, T., Lundberg, C., Kirik, D. & Wandless, T. J. A general chemical method to regulate protein stability in the mammalian central nervous system. *Chem Biol* **17**, 981-988, doi:10.1016/j.chembiol.2010.07.009 (2010).
