# Supplementary material for: SuperFi-Cas9 exhibits remarkable fidelity but severely reduced activity yet works effectively with ABE8e
Source: Nat Commun. 2022 Nov 11;13:6858. doi: 10.1038/s41467-022-34527-8 (PMC9652449; doi:10.1038/s41467-022-34527-8)
Supplement: Supplementary file 2 — Reporting Summary [file 41467_2022_34527_MOESM2_ESM.pdf]

nature portfolio

Corresponding author(s): Ervin Welker

Last updated by author(s): Oct 24, 2022

Reporting Summary

Nature Portfolio wishes to improve the reproducibility of the work that we publish. This form provides structure for consistency and transparency in reporting. For further information on Nature Portfolio policies, see our Editorial Policies and the Editorial Policy Checklist.

Statistics

For all statistical analyses, confirm that the following items are present in the figure legend, table legend, main text, or Methods section.

n/a	Confirmed	
☐	☒	The exact sample size (*n*) for each experimental group/condition, given as a discrete number and unit of measurement
☐	☒	A statement on whether measurements were taken from distinct samples or whether the same sample was measured repeatedly
☐	☒	The statistical test(s) used AND whether they are one- or two-sided<br>*Only common tests should be described solely by name; describe more complex techniques in the Methods section.*
☒	☐	A description of all covariates tested
☐	☒	A description of any assumptions or corrections, such as tests of normality and adjustment for multiple comparisons
☐	☒	A full description of the statistical parameters including central tendency (e.g. means) or other basic estimates (e.g. regression coefficient) AND variation (e.g. standard deviation) or associated estimates of uncertainty (e.g. confidence intervals)
☐	☒	For null hypothesis testing, the test statistic (e.g. *F*, *t*, *r*) with confidence intervals, effect sizes, degrees of freedom and *P* value noted<br>*Give P values as exact values whenever suitable.*
☒	☐	For Bayesian analysis, information on the choice of priors and Markov chain Monte Carlo settings
☒	☐	For hierarchical and complex designs, identification of the appropriate level for tests and full reporting of outcomes
☒	☐	Estimates of effect sizes (e.g. Cohen's *d*, Pearson's *r*), indicating how they were calculated

Our web collection on statistics for biologists contains articles on many of the points above.

Software and code

Policy information about availability of computer code

Data collection	Flow cytometry data collection was carried out on Attune NxT Acoustic Focusing Cytometer (Applied Biosystems) using Attune NxT Software v.4.2. Samples analyzed by NGS were sequenced on a MiniSeq or NextSeq (Illumina).
Data analysis	Targeted deep-sequencing data were analysed using a the following softwares: BBMap 38.08, samtools 1.8, BioPython 1.71, PySam 0.13. Indels were counted computationally amongst the aligned reads that matched at least 75% of the first 20bp of the reference amplicon. Indels without mismatches were searched starting at ±2bp around the cut site. For each sample, the indel frequency was determined as (number of reads with an indel) / (number of total reads). Frequency of substitutions without indels generated by base or prime editing was determined as the percentage of (sequencing reads with the intended modification, without indels) / (number of total reads). Allele frequency tables were generated using CRISPResso242. By contrast, frequency of intended insertions or deletions generated by prime editing was determined as the percentage of (all sequencing reads with only the intended insertions or deletions) / (number of total reads). For these samples the indel background was calculated from reads containing types of indels that were different from the aimed edit. The 15 bp long center fragment of the GUIDE-seq dsODN sequence (“gttgtcatatgttaa” / “ttaacatatgacaac”) was counted in the aligned reads to measure dsODN on-target tag integration for GUIDE-seq experiments. For FACS data analysis Attune NxT Software v.4.2 was used. Statistical tests were performed using GraphPad Prism 8. In case of in vitro data Origin 2018 was used.

For manuscripts utilizing custom algorithms or software that are central to the research but not yet described in published literature, software must be made available to editors and reviewers. We strongly encourage code deposition in a community repository (e.g. GitHub). See the Nature Portfolio guidelines for submitting code & software for further information.

Data

Policy information about availability of data

All manuscripts must include a data availability statement. This statement should provide the following information, where applicable:

- Accession codes, unique identifiers, or web links for publicly available datasets
- A description of any restrictions on data availability
- For clinical datasets or third party data, please ensure that the statement adheres to our policy

Expression vectors developed in this study are available from Addgene: pPIK16045_pX330_ Flag-SuperFi-Cas9 (#184370), pAT15542_nCBE-SuperFi-Cas9(#184372), pAT15544_ nABE-SuperFi-Cas9 (#184374), pAT15543_dABE-SuperFi-Cas9 (#184373), pAT15546_nABE8e-SuperFi-Cas9 (#184376), pAT15545_dABE8e-SuperFi-Cas9 (#184375), pAT15547_ PEmax-SuperFi-Cas9 (#184377). The deep sequencing data are available in NCBI Sequence Read Archive (accession number: PRJNA876837 [https://www.ncbi.nlm.nih.gov/bioproject/PRJNA876837/]).

Field-specific reporting

Please select the one below that is the best fit for your research. If you are not sure, read the appropriate sections before making your selection.

[x] Life sciences [ ] Behavioural & social sciences [ ] Ecological, evolutionary & environmental sciences

For a reference copy of the document with all sections, see nature.com/documents/nr-reporting-summary-flat.pdf

Life sciences study design

All studies must disclose on these points even when the disclosure is negative.

Sample size	No statistical methods were used to predetermine or justify sample size, but each condition was performed in tripliate which is generally accepted sample size for similar gene editing experiments.
Data exclusions	No data were excluded.
Replication	Independent replicates (n=3) were performed. All attempts at replication were successful.
Randomization	No randomization was necessary for the experimental design.
Blinding	No blinding was necessary, as no subjective assessments were required.

Reporting for specific materials, systems and methods

We require information from authors about some types of materials, experimental systems and methods used in many studies. Here, indicate whether each material, system or method listed is relevant to your study. If you are not sure if a list item applies to your research, read the appropriate section before selecting a response.

Materials & experimental systems

n/a	Involved in the study	
[ ]	[x]	Antibodies
[ ]	[x]	Eukaryotic cell lines
[x]	[ ]	Palaeontology and archaeology
[x]	[ ]	Animals and other organisms
[x]	[ ]	Human research participants
[x]	[ ]	Clinical data
[x]	[ ]	Dual use research of concern

Methods

n/a	Involved in the study	
[x]	[ ]	ChIP-seq
[ ]	[x]	Flow cytometry
[x]	[ ]	MRI-based neuroimaging

Antibodies

Antibodies used	anti-FLAG (F1804, Sigma); anti-β-actin (A1978, Sigma); HRP-conjugated secondary anti-mouse antibody (715-035-151, Jackson ImmunoResearch)

Validation	All the antibodies used in the study were validated by the manufacturer.<br>anti-FLAG (F1804, Sigma): https://www.sigmaaldrich.com/HU/en/product/sigma/f1804<br>anti-β-actin (A1978, Sigma): https://www.sigmaaldrich.com/HU/en/product/sigma/a1978<br>HRP-conjugated secondary anti-mouse antibody (715-035-151, Jackson ImmunoResearch): https://www.jacksonimmuno.com/catalog/products/715-035-151

Eukaryotic cell lines

Policy information about cell lines

Cell line source(s)	Cells employed in this study are HEK293 (Gibco 293-H cells); N2a.dd-EGFP (generated by us, for details see Methods section) N2a.EGFP (generated by us, for details see Methods section) and HEK-293.EGFP ((generated by us, for details see Methods section).
Authentication	Cell lines were not authenticated as they were obtained directly from a certified repository or cloned from those cell lines.
Mycoplasma contamination	Cells were tested monthly for mycoplasma contamination with negative test results.
Commonly misidentified lines (See ICLAC register)	No commonly misidentified cell lines were used.

Flow Cytometry

Plots

Confirm that:

- [x] The axis labels state the marker and fluorochrome used (e.g. CD4-FITC).
- [x] The axis scales are clearly visible. Include numbers along axes only for bottom left plot of group (a 'group' is an analysis of identical markers).
- [x] All plots are contour plots with outliers or pseudocolor plots.
- [x] A numerical value for number of cells or percentage (with statistics) is provided.

Methodology

Sample preparation	Cells were washed with PBS and after that trypsinized for 1 min. Completed DMEM was added after cells were separated (confirmed by microscope).
Instrument	Flow cytometry analysis was carried out using an Attune NxT Acoustic Focusing Cytometer (Applied Biosystems by Life Technologies).
Software	Attune Cytometric Software v.4.2
Cell population abundance	Viable single cells were gated based on side and forward light-scatter parameters and a total of 5,000 to 10,000 viable single cell events were acquired in all experiments.
Gating strategy	Negative controls (cells not transfected) were used to establish GFP, BFP and mCherry +/- gates. BFP, GFP and mCherry signals were detected using the 405 (for BFP), 488 (for GFP) and 561 nm (for mCherry) diode laser for excitation, and the 440/50 (BFP), 530/30 (GFP) and 620/15 (mCherry) filter for emission.

- [x] Tick this box to confirm that a figure exemplifying the gating strategy is provided in the Supplementary Information.
