# Supplementary material for: SuperFi-Cas9 exhibits remarkable fidelity but severely reduced activity yet works effectively with ABE8e
Source: Nat Commun. 2022 Nov 11;13:6858. doi: 10.1038/s41467-022-34527-8 (PMC9652449; doi:10.1038/s41467-022-34527-8)
Supplement: Supplementary file 3 — Description of Additional Supplementary Files [file 41467_2022_34527_MOESM3_ESM.pdf]

Title: Supplementary Data file 1:

Description: This file contains target, primer sequences and mutations of Cas9 variants.

Title: Supplementary Data file 2:

Description: This file contains EGFP disruption, BEAR and PEAR data.

Title: Supplementary Data file 3:

Description: This file contains NGS data.

Title: Supplementary Data file 4:

Description: This file contains GUIDE-seq data.

Title: Supplementary Data file 5:

Description: This file contains *in vitro* experiment data.

Title: Supplementary Data file 6:

Description: This file contains statistical details and exact p-values.

Title: Supplementary Data file 7:

Description: This file contains ABE allele table data.
